# Atheroprone fluid shear stress-regulated ALK1-Endoglin-SMAD signaling originates from early endosomes

**DOI:** 10.1186/s12915-022-01396-y

**Published:** 2022-09-28

**Authors:** Paul-Lennard Mendez, Leon Obendorf, Jerome Jatzlau, Wiktor Burdzinski, Maria Reichenbach, Vanasa Nageswaran, Arash Haghikia, Verena Stangl, Christian Hiepen, Petra Knaus

**Affiliations:** 1 Freie Universität Berlin, Institute for Chemistry and Biochemistry, Berlin, Germany; 2 Max Planck Institute for Molecular Genetics, Berlin, Germany; 3 International Max-Planck Research School for Biology and Computation, Berlin, Germany; 4 Berlin School for Regenerative Therapies, Berlin, Germany; 5 Charité–Universitätsmedizin Berlin, Klinik für Kardiologie, Campus Benjamin Franklin, Berlin, Germany; 6 DZHK (German Centre for Cardiovascular Research), Partner Site Berlin, Berlin, Germany; 7 Charité–Universitätsmedizin Berlin, Medizinische Klinik für Kardiologie und Angiologie, Campus Mitte, Berlin, Germany; 8 Berlin Institute of Health (BIH), Berlin, Germany; 9 Faculty of Engineering and Natural Sciences, Westphalian University of Applied Sciences, Recklinghausen, Germany

**Keywords:** Fluid shear stress, Endothelial cell, EndoMT, BMP, Endoglin, Caveolin, Endosome, Endocytosis, Atherosclerosis

## Abstract

**Background:**

Fluid shear stress enhances endothelial SMAD1/5 signaling via the BMP9-bound ALK1 receptor complex supported by the co-receptor Endoglin. While moderate SMAD1/5 activation is required to maintain endothelial quiescence, excessive SMAD1/5 signaling promotes endothelial dysfunction. Increased BMP signaling participates in endothelial-to-mesenchymal transition and inflammation culminating in vascular diseases such as atherosclerosis. While the function of Endoglin has so far been described under picomolar concentrations of BMP9 and short-term shear application, we investigated Endoglin under physiological BMP9 and long-term pathophysiological shear conditions.

**Results:**

We report here that knock-down of Endoglin leads to exacerbated SMAD1/5 phosphorylation and atheroprone gene expression profile in HUVECs sheared for 24 h. Making use of the ligand-trap ALK1-Fc, we furthermore show that this increase is dependent on BMP9/10. Mechanistically, we reveal that long-term exposure of ECs to low laminar shear stress leads to enhanced Endoglin expression and endocytosis of Endoglin in Caveolin-1-positive early endosomes. In these endosomes, we could localize the ALK1-Endoglin complex, labeled BMP9 as well as SMAD1, highlighting Caveolin-1 vesicles as a SMAD signaling compartment in cells exposed to low atheroprone laminar shear stress.

**Conclusions:**

We identified Endoglin to be essential in preventing excessive activation of SMAD1/5 under physiological flow conditions and Caveolin-1-positive early endosomes as a new flow-regulated signaling compartment for BMP9-ALK1-Endoglin signaling axis in atheroprone flow conditions.

**Supplementary Information:**

The online version contains supplementary material available at 10.1186/s12915-022-01396-y.

## Background

Hemodynamic forces have been shown to play a key role in the development of vascular diseases including atherosclerosis, thrombosis, and aneurysms [1]. Of these, perturbed shear stress (SS) is recognized as one of the main biomechanical factors for vascular disease development and progression [2]. Low SS (LSS) (< 4 dyn/cm^2^) often found with oscillatory or disturbed hemodynamic patterns is present at the outer edges of arterial bifurcations or the lesser aortic curvature and leads to endothelial cell (EC) activation and dysfunction, ultimately resulting in an atheroprone phenotype [3, 4]. In contrast, high laminar SS (HSS) (> 15 dyn/cm^2^), which occurs in straight parts of the arterial vasculature, is protective and maintains ECs in a quiescent state [4]. Along with hemodynamic forces, perturbed biochemical signals including growth factors of the transforming growth factor beta (TGFβ)/bone/body morphogenetic protein (BMP) signaling axis have been shown to regulate the transition of ECs from an endothelial towards a mesenchymal cell fate (EndoMT) [5]. Thereby, a combination of perturbed TGFβ/BMP signaling and biomechanics is contributing to the pathogenesis of atherosclerosis [6] and EC inflammation [7, 8].

BMP9/10 are soluble, systemic ligands which are circulating in the blood stream, where they act on the endothelium at picomolar concentrations. They constantly provide survival and quiescence signals for ECs via binding to high-affinity type I receptor Activin-like kinase 1 (ALK1) together with type II receptors and facilitated by co-receptor Endoglin [9, 10]. Endoglin efficiently captures soluble BMP9 from the blood at the EC surface allowing for non-competitive binding of the type I receptor. Subsequently, Endoglin is replaced by the type II receptor to create a hetero-tetrameric receptor signaling complex [11]. Regulation of target genes via canonical SMAD1/5 transcription factor signaling is context dependent as BMP9 alone regulates the expression of inhibitor of differentiation (*ID*) genes, or in conjunction with other factors like tumor necrosis factor alpha (TNF-α), the expression of *SELE* [12] or *EDN1* [13]. De-regulated BMP signaling drives EC proliferation, de-differentiation, and an inflammatory gene signature [14, 15]. BMP signaling thus requires fine-tuning to be maintained at optimal levels by means of magnitude and intensity in a spatiotemporal manner. How protective high SS versus atheroprone low SS integrates into the regulation of the ALK1-Endoglin-SMAD1/5 signaling axis is only little understood.

In the past years, the molecular mechanisms explaining the integration of biomechanics into BMP/TGFβ signaling have been expanding [16], particularly since it was shown that BMP signaling is over-activated at sites of atherosclerotic lesions in humans, mice, and rats [17]. This was further underlined by experiments in which inhibiting BMP-SMAD1/5 signaling reduces vascular calcification and atherosclerosis in LDLR^−/−^ or ApoE^−/−^ mice [14, 18]. Since then, several studies addressed the role of different hemodynamics in fine-tuning and balancing BMP9/10-induced SMAD signaling. SS was shown to sensitize ECs to BMP9 ligands via Endoglin, suggesting a flow-induced complex formation between BMP receptors and co-receptors at the EC plasma membrane [19] similar to previous data showing a flow-induced oligomerization of BMP receptors and integrins [20]. Additionally, both *Acvrl1* (encoding for Alk1) [21] and *Eng* (encoding for Endoglin) [22] have been described to be shear-sensitively transcribed in the murine vasculature, adding a second layer of regulation. Beyond, further mechanisms focusing on spatiotemporal regulation of ALK1-Endoglin complexes, their fates after shear exposure, and relative distribution are virtually lacking particularly in the context of EC long-term adaptation to low vs. high fluid SS (FSS).

BMP receptor endocytosis occurs via different routes and represents a prime paradigm of fine-tuning and balancing BMP signaling outcomes as shown by us and others [23–27]. A widely accepted model for TGFβ receptor endocytosis states that Clathrin-mediated endocytosis (CME) induces SMAD signaling while Caveolin-mediated endocytosis (CvME) leads to a termination of signaling and degradation of receptors [28]. Caveolin-1-positive rafts on the other hand were found to be enriched in SMAD7 and Smurf2, both signaling modulators competing with SMAD activation or priming TGFβ receptors for degradation, respectively [28]. Recently, an additional model was proposed being that both CME and CvME converge into early endosome antigen 1 (EEA-1)-positive EEs during endocytosis of TGFβ type I receptors. Importantly, these EEs serve as regulatory signaling hotspots for TGFβ-SMAD2/3 signaling [29]. If this finding would be conserved also for BMP-SMAD1/5 signaling, it could provide a mechanism of how CvME could switch from an antagonist to a promoter of BMP-SMAD signaling at the level of internalized EEs and at the crossroads with CME.

We now show that atheroprotective HSS protects from while atheroprone LSS favors the accumulation of ALK1 and Endoglin in Caveolin-1-positive EEs, which we herein identify to serve as signaling hotspots initiating atheroprone SMAD1/5 signaling in ECs. In this context, depletion of Caveolin-1 via siRNA-mediated knock-down leads to reduced SMAD1/5 activation providing a new mode of how hyperactive BMP signaling could be targeted in the vasculature. Furthermore, we demonstrate that Endoglin possesses a protective role in the vascular endothelium by preventing excessive BMP9-ALK1 signaling and subsequent expression of *EDN1*.

## Results

### Validation of the flow system

Mimicking different human hemodynamics in vitro is technically challenging. We thus aimed for a simplistic approach, exposing ECs cultured in 2D to defined laminar shear stress (SS) recapitulating the physical hallmarks of two distinct flow regimes, low atheroprone and high atheroprotective laminar SS (LSS and HSS, respectively).

**Fig. 1:**
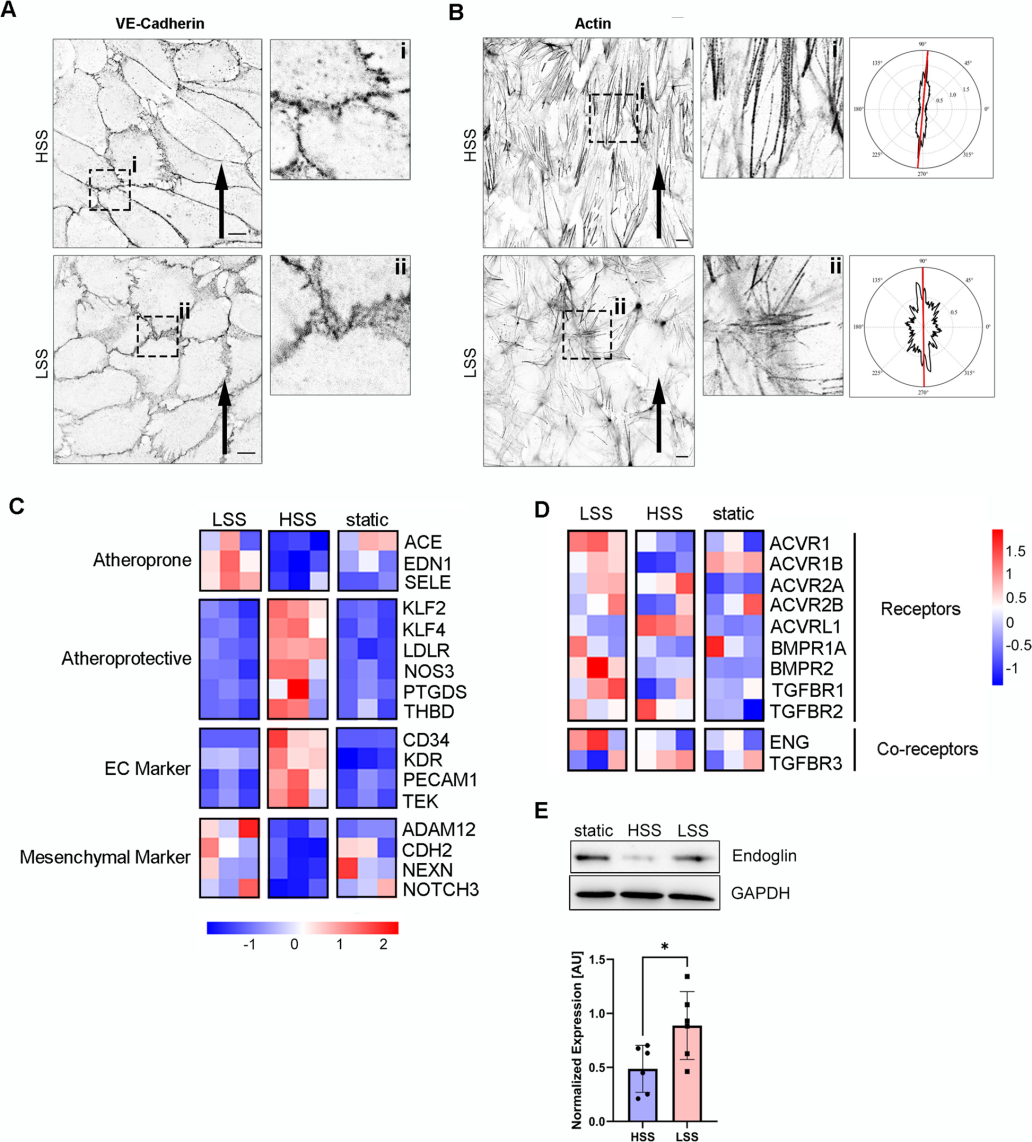
High and low FSS lead to differential adaption of ECs and changes in BMP receptor expression. **A** Confocal images of VE-Cadherin staining. Insets show magnified regions. **B** Confocal images of Actin fiber staining by phalloidin. Insets show magnified regions. Polar plot of normalized power intensity that plots the mean actin fiber orientation (red line) using an elliptical best fit. Arrows in **A** and **B** indicate the direction of flow application. Scale bars 2 μM. **C** Heatmap of selected marker genes from RNAseq data set. All selected genes are differentially expressed (adjusted *p*-value < 0.05, fold change >1.5) between LSS and HSS. **D** Expression of BMP receptors and co-receptors from RNAseq data, independent of their adjusted *p*-value or fold change. Color-coding for **C** and **D** shows *z*-scores (red = high, blue = low). **E** Immunoblot using antibodies specific against Endoglin (upper panel). Densiometric quantification of Endoglin expression relative to GAPDH (lower panel). Data is presented as mean ± SD from 6 independent experiments. Expression was normalized to statical control. Statistical significance was calculated by unpaired, two-sided Student’s *T*-test, *p < 0.05

To validate this approach, we first evaluated whether human umbilical vein ECs (HUVECs) exposed to LSS (1 dyn/cm^2^) or HSS (30 dyn/cm^2^) exhibit previously reported morphological adaptations of the cell’s VE-Cadherin-rich cell junctions and concomitant alignment of filamentous actin (F-actin) cytoskeleton. For this, we subjected ECs to the mentioned flow regimes for 24 h and observed that cells exposed to LSS showed broader and less confined VE-Cadherin distribution at the cell junctions (Fig. 1A), indicative for EC dysfunction as demonstrated before [30–32]. Moreover, exposure to HSS favored F-actin stress fiber alignment with the direction of flow and an overall increase of elongated cells, when compared to LSS (Fig. 1B). This is in line with reported hallmarks of long-term flow adaptation [33]. Next, we investigated whether LSS and HSS led to distinct transcriptional adaptations reflecting major markers of an atheroprone or atheroprotective EC phenotype. We performed RNA sequencing analysis (RNAseq, see Additional files 2 and 3 for data, raw data available from GSE211662 [34]) of HUVECs exposed to HSS or LSS for 24 h screened for differentially expressed genes (DEGs, adjusted *p*-value < 0.05, log_2_FC > |0.585|) and observed a total number of 2713 (1450 up, 1263 down) and 656 (228 up, 428 down) DEGs in HSS vs. static and LSS vs. static, respectively (Additional file 1: Fig. S1 A/B). Next, we performed gene ontology (GO) analysis using DAVID database [35] and observed a contrary pattern of terms in DEGs from HSS vs. static and LSS vs. static. In HSS, genes corresponding to GO terms cell division, DNA replication, mitotic nuclear division, sister chromatid cohesion, and G1/S transition of mitotic cell cycle were downregulated while upregulated in LSS (Additional file 1: Fig. S1 C/D). This indicates that HSS induces quiescence while LSS leads to a proliferative state of HUVECs. Detailed analysis of the RNAseq data revealed that LSS upregulated atheroprone genes, indicative for EC dysfunction, such as *EDN1*, *ACE*, and *SELE* [36–41], while atheroprotective genes *NOS3*, *KLF4*, *KLF2*, *LDLR*, *THBD*, and *PTGDS* [42] were induced by HSS (Fig. 1C). In addition, endothelial markers were upregulated upon HSS application while mesenchymal markers were upregulated only upon exposure of ECs to LSS (Fig. 1C) [6]. To validate that regulation of marker genes is similar in ECs from a different vascular bed, we performed a qPCR analysis of human aortic ECs (HAoECs) and observed regulation of crucial genes *KLF2*, *TEK*, *CD34*, and *EDN1* comparable to HUVECs (Additional file 1: Fig. S1E). To prove that our laminar SS application approach reflects on published SS-regulated DEGs, we compared our data to publicly available datasets [41] (GSE103672 [43]). Importantly, we found that differential gene expression from our mechanical model recapitulates differential gene expression induced by more complex oscillatory (OS) or pulsatile (PS) flow as hierarchical clustering analysis clustered HSS/PS and LSS/OS together (Additional file 1: Fig. S2A). Furthermore, gene expression profile was similar between HSS and PS or LSS and OS (Additional file 1: Fig. S2A) and LSS/HSS compared with OSS/PS showed a Pearson correlation coefficient of *R* = 0.87 (Additional file 1: Fig. S2B). Moreover, detailed analysis showed that expression of atheroprone/atheroprotective, EC/mesenchymal genes depicted in Fig. 1C are regulated accordingly in OSS/LSS and HSS/PS (Additional file 1: Fig. S2C). This proves that our SS model is suitable to study flow responses under physical conditions with relevance for mechanically more complex scenarios.

### BMP receptor expression in LSS- and HSS-exposed HUVECs

Next, we used the RNAseq data to analyze the expression of BMP receptors and relevant co-receptors. Interestingly, *ACVRL1* was upregulated under HSS while the majority of BMP and co-receptors were upregulated under LSS, including the ALK1 co-receptor Endoglin (*ENG*) (Fig. 1D). We continued with analyzing protein levels of Endoglin and found that indeed Endoglin levels were significantly lower in HSS compared to LSS upon EC exposure to 24-h laminar flow (Fig. 1E). Additionally, we performed the same experiment in the presence of 20% human serum instead of fetal calf serum and found that Endoglin was similarly upregulated in LSS compared to HSS (Additional file 1: Fig. S3A).

### Long-term induction of BMP signaling and upregulation of atheroprone genes in LSS is BMP9/10 dependent

**Fig. 2:**
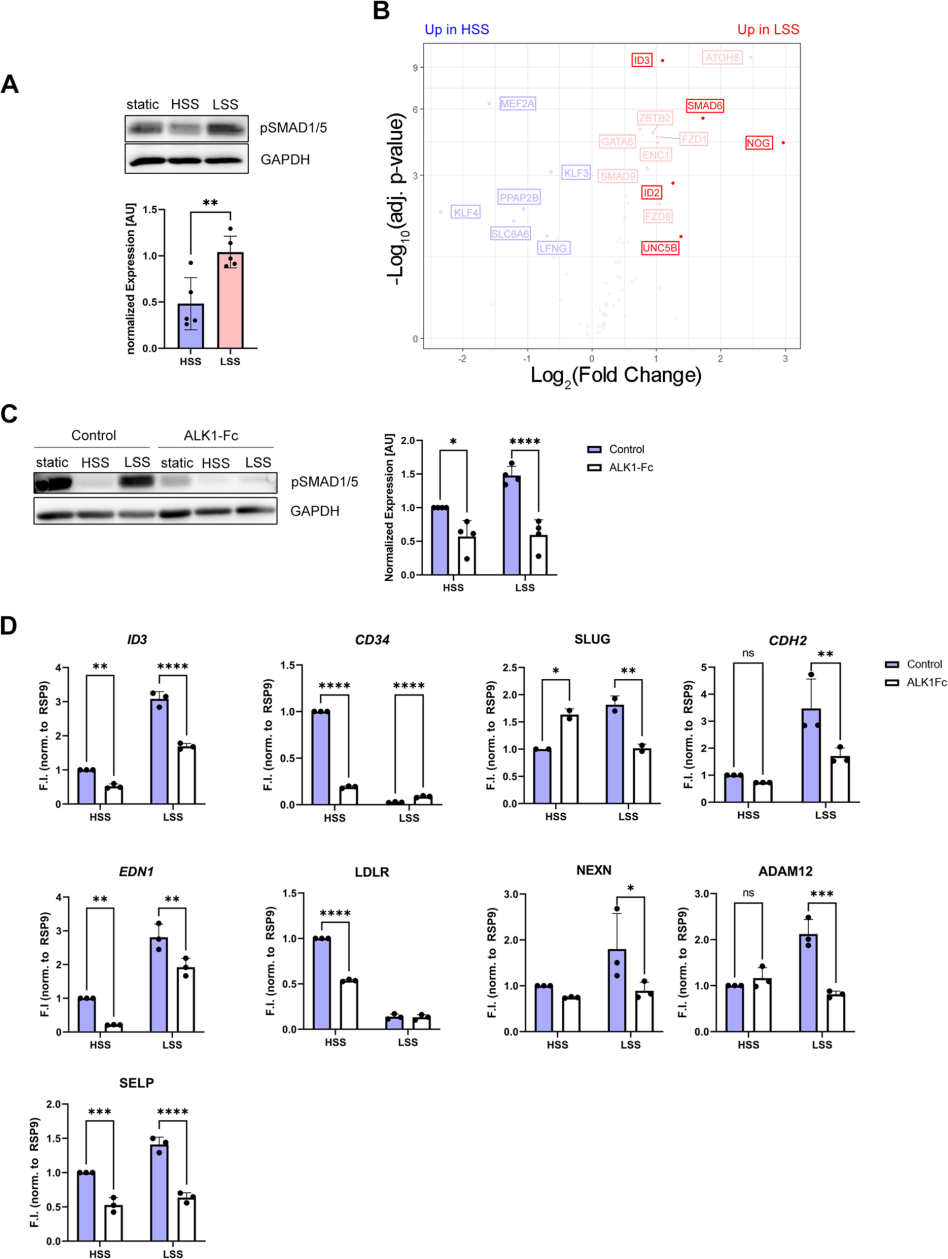
Differential impact of HSS and LSS on ALK1-mediated BMP signaling. **A** Immunoblot using antibodies specific against pSMAD1/5 shows different levels of SMAD1/5 phosphorylation in HSS versus LSS after 24 h of FSS exposure (upper panel). Densiometric quantification of SMAD1/5 phosphorylation relative to GAPDH expression. Data is presented as mean ± SD from 5 independent experiments. Statistical significance against static control was calculated by unpaired, two-sided Student’s *T*-test. **B** Volcano plot depicting –log_10_(adjusted *p*-value) against log_2_(fold change) from RNAseq data of BMP target genes compiled from [45]. Highlighted genes are commonly used BMP target genes. Red indicates upregulation in LSS, and blue indicates upregulation in HSS. Genes depicted in gray are not significantly regulated. **C** Immunoblot using antibodies specific against pSMAD1/5 showing responses of 24-h exposure to FSS in the absence/presence of 50 ng/mL ALK1-Fc (left panel). Densiometric quantification of SMAD1/5 phosphorylation relative to GAPDH expression. Data is presented as mean ± SD from 4 independent experiments. Statistical significance against HSS control was calculated using 2-way ANOVA and Šídák’s post hoc test (right panel). **D** Quantitative PCR showing gene expression of selected markers after 24-h FSS application in the absence/presence of 50 ng/mL ALK1-Fc. Data is presented as mean ± SD from 3 independent experiments. Statistical significance against HSS control was calculated using 2-way ANOVA and Šídák’s post hoc test, ****p < 0.0001,***p < 0.001, **p < 0.01, *p < 0.05

Previous work has shown that atheroprone oscillatory SS (OSS) induces sustained activation of SMAD1/5 [17]. While it was stated that this long-term induction of SMAD1/5 phosphorylation in OSS versus LSS was independent of BMP ligands [17], more recent studies showed that short-term laminar SS-mediated induction of SMAD1/5 phosphorylation under low-serum conditions was dependent on BMP9/10 [19, 44]. Along that line, we here show that long-term (24 h) application of LSS increased SMAD1/5 phosphorylation when compared to HSS in the presence of FCS (Fig. 2A) or human serum (Additional file 1: Fig. S3A). In consequence, BMP target gene transcription was elevated in LSS over HSS (Fig. 2B). Furthermore, we observed that this induction was dependent on ALK1 ligands, presumably BMP9 and BMP10, since treatment with 50 ng/mL ALK1-Fc, an established ligand-trap for BMP9 and BMP10 [46], abolished this response significantly (Fig. 2C). Accordingly, expression of BMP target gene *ID3*, as well as inflammatory marker *EDN1*, adhesion molecule P-Selectin (*SELP*) [47] and mesenchymal markers *CDH2*, *ADAM12*, *NEXN*, and EndoMT mediating transcription factor *SLUG* [48] were reduced in HUVECs exposed to LSS and treated with ALK1-Fc (Fig. 2D). Noteworthy, the expression of venous EC marker *CD34* and atheroprotective *LDLR* were decreased in HSS after application of ALK1-Fc (Fig. 2D). To further validate the dependency of crucial genes on BMP receptor signaling, we performed inhibitor treatment with LDN-193189 (LDN), which blocks BMPRI kinase activity [49]. We added 1.8 μM LDN to HUVECs exposed to LSS which led to a reduction of pSMAD1/5 levels to that of HSS-exposed HUVECs (Additional file 1: Fig. S3B). Subsequent analysis of *CDH2* and *EDN1* expression revealed treatment with 1.8 μM LDN leads to equal expression levels in LSS and HSS control (Additional file 1: Fig. S3C). Taken together, these data show that the induction of SMAD1/5 phosphorylation and the subsequent expression of *EDN1* and especially mesenchymal genes upon LSS exposure is highly dependent on BMP9/10. Nevertheless, BMP-ALK1 signaling together with HSS is crucial for the expression of important vascular protective genes maintaining the endothelial phenotype. However, we additionally found inflammatory and EC markers (*ACE*, *CDH5*, and *TEK*, respectively) that were regulated by SS but not BMP signaling (Additional file 1: Fig. S3D). This highlights that BMP signaling integrates into SS-mediated transcriptional changes for a specific yet important set of target genes.

### Endoglin is necessary for acute flow induction of pSMAD1/5 only in low serum concentrations

**Fig. 3:**
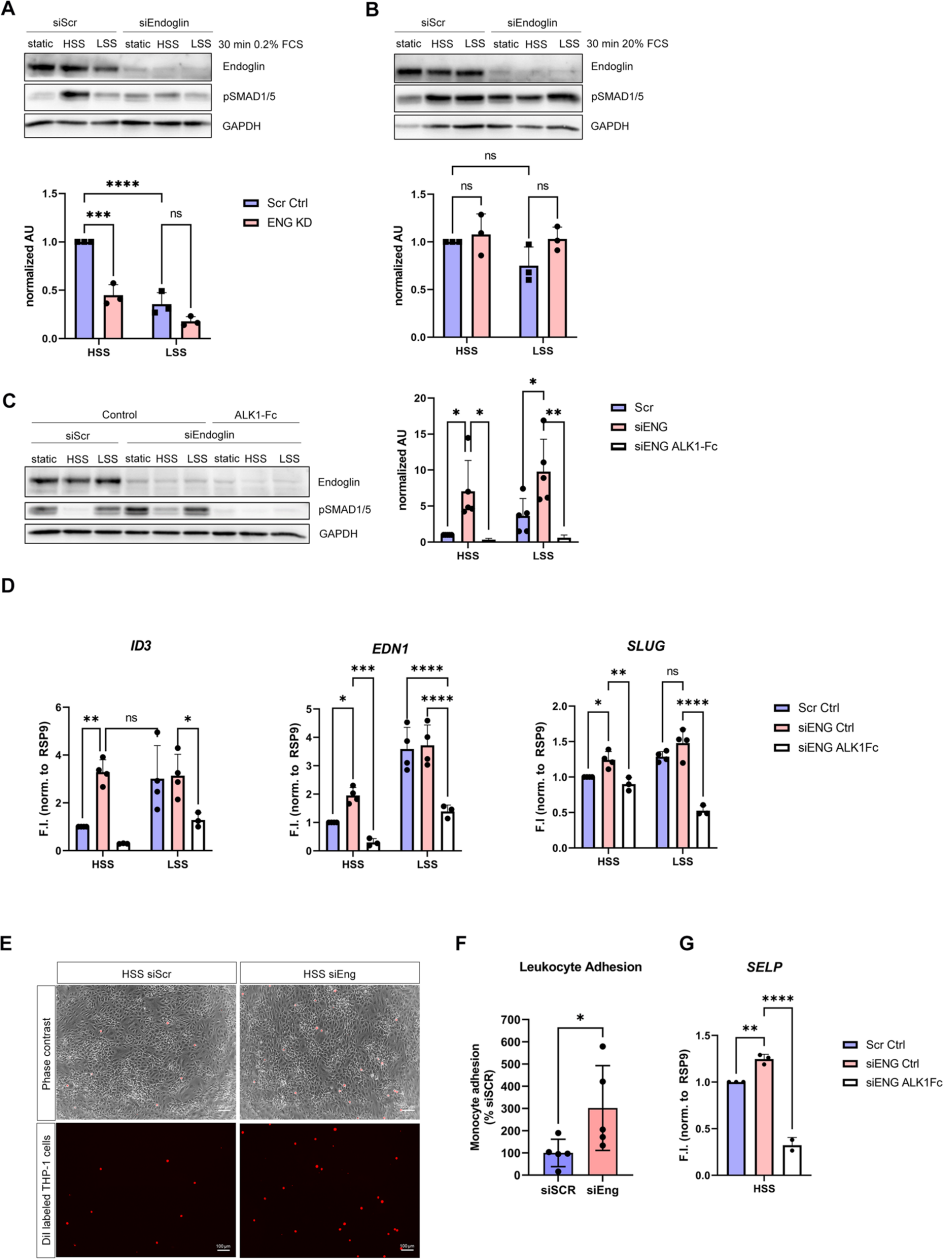
Endoglin regulates FSS-induced, ALK1-mediated BMP signaling depending on serum concentration. **A**, **B** Immunoblots using antibodies specific against pSMAD1/5 and Endoglin in cells exposed to 30 min of FSS in presence of **A** 0.2% or **B** 20% FCS after Endoglin knock-down or scrambled siRNA controls (upper panels). Densiometric quantifications of SMAD1/5 phosphorylation relative to GAPDH expression (lower panels). Data is presented as mean ± SD from 3 independent experiments. Statistical significance against HSS control was calculated using 2-way ANOVA and Šídák’s post hoc test. Statistical significance in between groups (HSS vs. LSS) was calculated using 2-way ANOVA and Šídák’s post hoc test, ns means *p* >0.05. **C** Immunoblot using antibodies specific against pSMAD1/5 and Endoglin in cells exposed to 24 h of FSS after Endoglin knock-down or scrambled siRNA controls in the absence/presence of 50 ng/mL ALK1-Fc (left panel). Densiometric quantification of SMAD1/5 phosphorylation relative to GAPDH expression (right panel). Data is presented as mean ± SD from 3–5 independent experiments. **D** Quantitative PCR showing gene expression of selected markers after 24-h FSS application in Endoglin knock-down or scrambled siRNA control cells in the absence/presence of 50 ng/mL ALK1-Fc. Data is presented as mean ± SD from 4 independent experiments. Statistical significance was calculated using 2-way ANOVA and Šídák’s post hoc test, ****p < 0.0001,***p < 0.001, **p < 0.01, *p < 0.05. **E** Phase-contrast and immunofluorescence images of leukocyte adhesion assay on HUVECs transfected with siRNA against Endoglin or scrambled control and subjected to HSS for 24h. **F** Quantification of leukocyte (THP-1) cell adhesion shown in **E**. Data is shown as percentage of mean scrambled control adhesion. Statistical significance was calculated using Mann-Whitney test. *p<0.05. **G** Quantitative PCR showing gene expression of SELP after 24-h HSS application in Endoglin knock-down or scrambled siRNA controls cells in the absence/presence of 50 ng/mL ALK1-Fc. Statistical significance against HSS control was calculated using 2-way ANOVA and Šídák’s post hoc test, ****p < 0.0001,***p < 0.001, **p < 0.01, *p < 0.05

To extend on a previous study which demonstrated that Endoglin was only necessary to promote BMP9-induced phospho-Smad1/5 signaling in the presence of FSS [19], we investigated whether Endoglin’s role in committing BMP-SMAD responsiveness to short FSS application is seen under both low (0.2% FCS) and high (20% FCS) serum concentrations following a short-term (30 min) FSS pulse. For this, we performed siRNA-mediated knock-down and exposed Endoglin-depleted cells to 30 min of LSS and HSS in the presence of 0.2% FCS. We could recapitulate that acute flow induction of pSMAD1/5 is dependent on the magnitude of the force applied (HSS > LSS) and on the presence of Endoglin (Fig. 3A). However, when we performed the same experiment without limiting the serum concentration (20% FCS), knock-down of Endoglin did not show an effect on SMAD1/5 phosphorylation (Fig. 3B) identifying Endoglin to be required for integrating acute FSS responses towards SMAD signaling only in the presence of low serum, i.e., limited concentrations of BMP9/10.

### Endoglin protects from excessive pSMAD1/5 response in flow-adapted ECs

Strikingly, in the presence of 20% serum and long-term (24 h) FSS exposure, we found that Endoglin depletion markedly increased phospho-SMAD1/5 levels in both, LSS and HSS conditions (Fig. 3C). Thus, we conclude that while Endoglin is necessary for FSS-enhanced BMP9/10 signaling in serum conditions of ligand scarcity, it is dispensable under ligand saturation. Furthermore, in FSS-adapted HUVECs, Endoglin unexpectedly appears to limit the BMP9/10-ALK1-phospho-SMAD1/5 response. This clearly indicates that Endoglin is particularly important under conditions, in which ALK1 or its ligands are limited (e.g., genetically primed ALK1 or BMP9/10 deficiencies which lead to severe vascular pathologies like hereditary hemorrhagic telangiectasia (HHT) or pulmonary arterial hypertension (PAH) [50–52]), while it appears to adopt a negative regulatory role for phospho-SMAD1/5 signaling upon EC adaptation to flow.

Additionally, we analyzed the expression of *ID3*, *EDN1*, and *SLUG* and found that their expression was enhanced under HSS and concomitant Endoglin knock-down versus control condition (Fig. 3D), suggesting a protective role of Endoglin for flow-adapted ECs. Nonetheless, BMP9/10-dependent mesenchymal genes *CDH2*, *ADAM12*, and *NEXN* were not regulated by Endoglin KD in HSS (Additional file 1: Fig. S4A). Endothelial marker *CD34* and atheroprotective *LDLR*, however, were both upregulated upon Endoglin KD in HSS (Additional file 1: Fig. S4A).

To further characterize the functional outcome of Endoglin KD, we performed a leukocyte adhesion assay on control or Endoglin KD cells exposed to HSS. We could show that upon Endoglin KD significantly more leukocytes adhere to the HUVEC monolayer compared to the control condition (Fig. 3E, F). This is in line with the enhanced expression of *SELP* in the KD condition (Fig. 3G).

Next, to elucidate whether the exacerbated SMAD1/5 phosphorylation upon Endoglin knock-down (KD) is dependent on BMP9/ALK1 signaling, we performed Endoglin knock-down experiments in the presence of 50 ng/mL ALK1-Fc. We found that the addition of ALK1-Fc effectively abolished SMAD1/5 phosphorylation and lowered target gene expression below levels of the scrambled control (Fig. 3C, D). Therefore, we conclude that Endoglin is necessary to protect ECs from acquiring an atheroprone phenotype and that the increase in SMAD1/5 phosphorylation upon loss of Endoglin is dependent on BMP9/10.

### Identification of Caveolin-rich early endosomes as a new compartment for LSS-induced BMP signaling

We have shown that our FSS application is sufficient to generate either atheroprotective or atheroprone long-term phospho-SMAD1/5 responses via ALK1-ENG signaling. However, the molecular mechanisms leading to LSS-induced increases in SMAD1/5 phosphorylation beyond the levels found under protective HSS, remain elusive. While others suggested a cooperative engagement of integrin signaling into FSS-induced BMP-SMAD signaling [20], alternative mechanisms of how FSS integrates into the regulation of SMAD1/5 phosphorylation are lacking. It has been recently reported that ALK5, a type I TGFβ receptor, is internalized both via Caveolin-1- and Clathrin-dependent endocytosis and localizes to Caveolin-1-positive EEs and that these endosomes are enriched for signaling components of the TGFβ pathway [29]. Interestingly, also ALK1 [53] and Endoglin [54] were shown to localize to Cav-1 membrane domains. Cell surface-associated caveolae may act as FSS sensors and participate in receptor-mediated mechanotransduction [55, 56]. Moreover, it was demonstrated that knock-down of Caveolin-1 decreases BMP9-induced SMAD1/5 phosphorylation in HUVECs cultivated under static conditions [57]. We therefore hypothesized that Caveolin-1 is part of a regulatory network of flow-induced BMP9-Endoglin-ALK1 signaling.

**Fig. 4:**
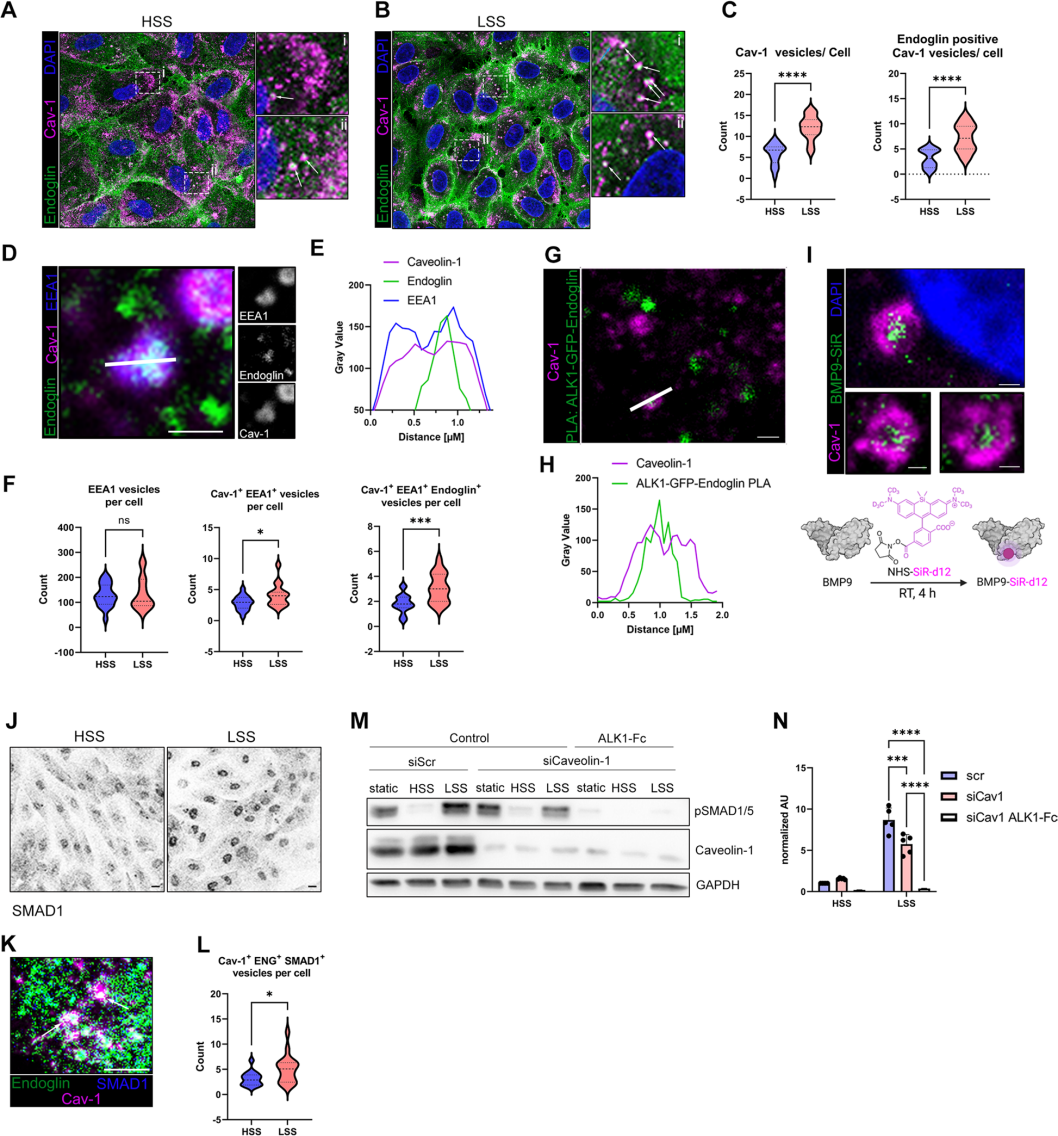
Caveolin-1-positive early endosomes are signaling hotspots for FSS-induced BMP signaling. Confocal images of Endoglin, Caveolin-1, and DAPI in cells exposed to **A** HSS or **B** LSS. Insets show magnified regions. Arrows indicate Endoglin-positive Caveolin vesicles. Scale bars 10 μM. **C** Quantification of the number of Caveolin-1 vesicles (left) and Endoglin-positive Caveolin-1 vesicles (right). Counting was performed using a self-written ImageJ script (see the “Methods” section for details). **D** Confocal image of Endoglin, Caveolin-1, and EEA1. Scale bar 1 μM. Insets show single channels in gray scale. The white line indicates the area of intensity profile shown in **E**. **E** Intensity profile of Endoglin, Caveolin-1, and EEA1 staining shown in **D**. **F** Quantification of EEA1 vesicles (left), Cav-1-positive EEA1 vesicles (middle), and Cav-1 and Endoglin double-positive EEA1 vesicles (right). **G** Confocal image of Caveolin-1 and PLA of Endoglin and overexpressed ALK1-meGFP. Scale bar 1 μM. The white line indicates the area of intensity profile shown in **H**. **H** Intensity profile of Caveolin-1 and Endoglin-ALK1-meGFP PLA staining shown in **G**. **I** Confocal image of Caveolin-1, deuterated silicon-rhodamine-labeled BMP9 (BMP9-siR), and DAPI. Scale bars 500 nm. The lower panel schematically depicts the labeling process of BMP9 with deuterated silicone-rhodamine (see the “Methods” section). **J** Inverted confocal images of SMAD1 show the nuclear accumulation of SMAD1 in LSS over HSS. Scale bar is 10 μM. **K** Confocal image of Caveolin-1, Endoglin, and SMAD1 shows the location of SMAD1 at Endoglin-positive Caveolin-1 vesicles, indicated by arrows. Scale bar 5 μM. **L** Quantification of SMAD1-positive Endoglin/Cav-1 vesicles. **M** Immunoblot using antibodies specific against pSMAD1/5 and Caveolin-1 in cells exposed to 24 h of FSS after Caveolin-1 knock-down in the absence/presence of 50 ng/mL ALK1-Fc. **N** Densiometric quantification of SMAD1/5 phosphorylation relative to GAPDH expression from immunoblots in **M**. Data is presented as mean ± SD from 3–5 independent experiments. Statistical significance against HSS control was calculated using 2-way ANOVA and Šídák’s post hoc test, ****p < 0.0001,***p < 0.001

Thus, we analyzed next the distribution of Caveolin-1 and Endoglin upon application of long-term HSS and LSS. We observed a high number of intracellular vesicles rich in Endoglin and positive for Caveolin-1 (Fig. 4A, B, indicated by arrows). Interestingly, the number of Caveolin-rich vesicles and those concomitantly positive for Endoglin were significantly higher upon exposure to LSS compared to HSS (Fig. 4C). Co-staining of Caveolin-1, Endoglin, and EEA1 identified these vesicles as EEs (Fig. 4D, E). We analyzed whether there were differences in the occurrence of Cav-1 EEA1-positive vesicles between HSS and LSS and found that there were significantly more EEA1-positive Cav-1 vesicles in LSS compared to HSS (Fig. 4F). This was confirmed in HAoECs, highlighting that the adaptation of ECS to FSS is independent of their vascular bed (Additional file 1: Fig. S5 A/B). Next, we analyzed whether both, Endoglin and ALK1, are present in the Caveolin-1-positive EEs and performed a proximity ligation assay (PLA) for overexpressed ALK1-GFP with endogenous Endoglin and co-stained for Caveolin-1. Here, we observed PLA signals inside of Caveolin-1-positive vesicles indicating that the ALK1-Endoglin receptor complexes are locating in the EE compartment (Fig. 4G, H). Furthermore, we conjugated BMP9 with deuterated silicon-rhodamine (SiR) [58, 59] to obtain labeled BMP9 ligand. Co-staining with Caveolin-1 revealed the presence of SiR-labeled BMP9 in Caveolin-1 vesicles (Fig. 4I). Together, this clearly indicates that active, ligand-bound receptor signaling complexes are present in Caveolin-1-rich early endosomes. To further prove that these complexes are actively engaged in SMAD signaling, we performed a co-staining of SMAD1 and Caveolin-1. Indeed, we observed accumulation of SMAD1 at sites of Caveolin-1-rich vesicles (Fig. 4K) concomitant with a significantly higher fraction of SMAD1/Endoglin-positive Cav-1 vesicles in LSS when compared to HSS (Fig. 4L). Additionally, as expected from published results, we found a strong accumulation of SMAD1 in the nucleus of LSS-treated ECs (Fig. 4J) [17, 19]. Again, we validated the presence of SMAD1/Endoglin-positive Caveolin-1 vesicles in HAoECs and show that more Endoglin and Endoglin/SMAD-positive Caveolin vesicles are present in LSS (Additional file 1: Fig. S5 C/D). Taken together, we demonstrate that Endoglin, together with ALK1 and BMP9, localizes to Caveolin-1-rich EEs, which we identify here as a new yet unrecognized signaling compartment that facilitates SMAD accumulation under long-term LSS exposure in ECs.

To finally prove that Caveolin-1 is indeed necessary for elevated BMP signaling under LSS exposure, we performed Caveolin-1 knock-down experiments. Depletion of Caveolin-1 reduced the LSS-dependent phospho-SMAD1/5 levels in HUVECs (Fig. 4M/N). Addition of ALK1-Fc confirmed that the remaining active SMAD1/5 originated still from ALK1-dependent signaling complexes (Fig. 4M/N).

## Discussion

**Fig. 5:**
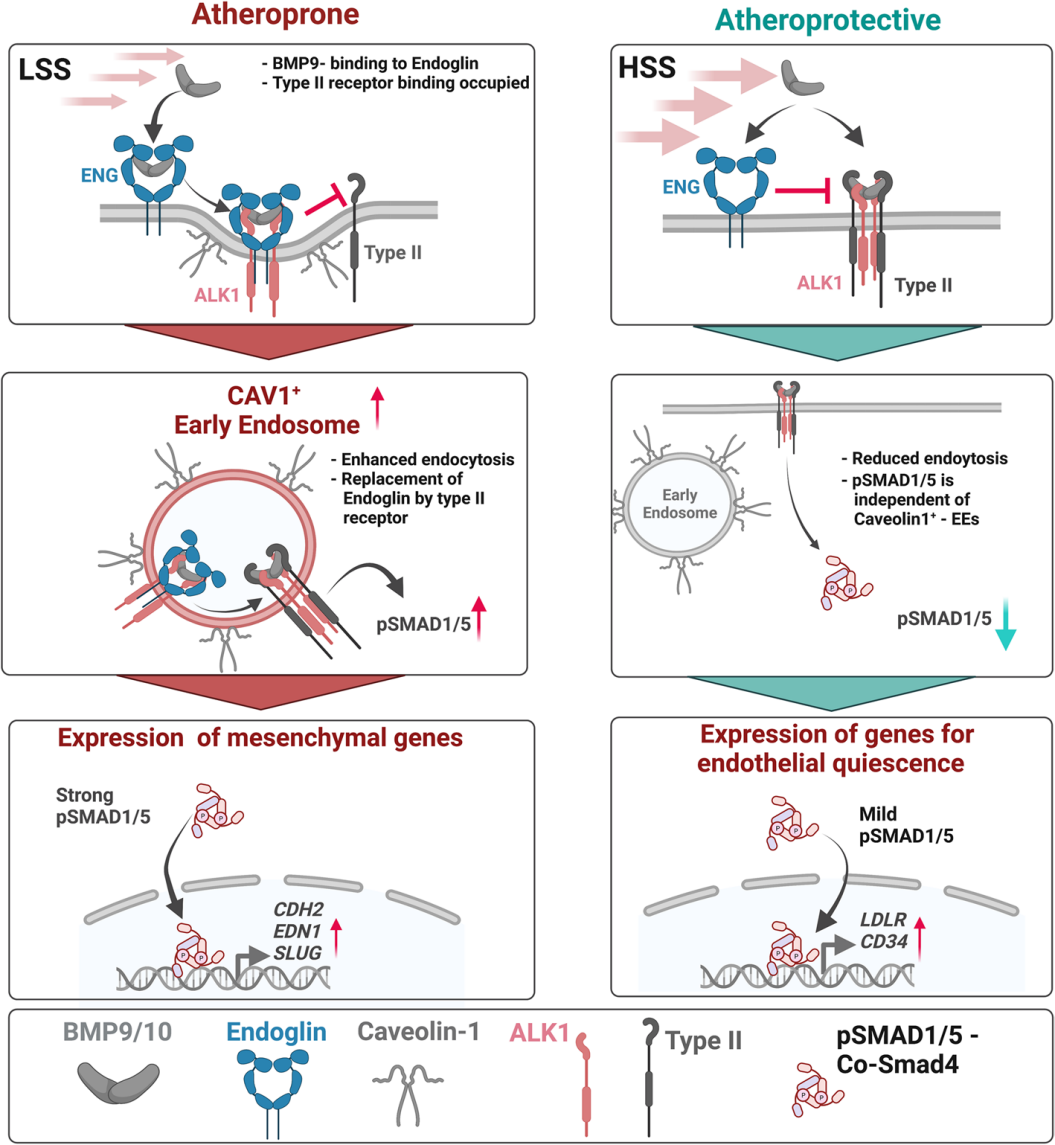
Summary scheme. Under atheroprone LSS conditions (left), circulating BMP9 gets sequestrated by Endoglin, expressed on endothelial cells. Endoglin binds to the type II receptor interface, thereby blocking BMP type I/II receptor complex formation. Nonetheless, residual BMP9-ALK1-type II receptor signaling occurs at the plasma membrane. Upon internalization via caveolae, Endoglin gets substituted by type II receptors in EEs and exacerbated pSMAD1/5 signaling is induced which leads to the expression of mesenchymal and atheroprone genes. Under atheroprotective HSS conditions (right), BMP9 is bound by ALK1 and type II receptors as well as by Endoglin, the latter suppressing BMP9-type I/II receptor formation. Caveolin-mediated endocytosis is markedly reduced when compared to LSS and residual pSMAD1/5 signaling is originating from the plasma membrane rather than from CAV1^+^ EEs. Ultimately, HSS leads to the expression of genes responsible for EC identity and quiescence

Blood flow and in particular the mechanical FSS acting on the surface of ECs are known inducers and sensitizers of the BMP9-ALK1-Endoglin-SMAD1/5 signaling axis. In this context, it was shown that Endoglin is dispensable for BMP9-mediated SMAD1/5 activation under static culture conditions but indispensable for FSS-mediated SMAD1/5 activation [19]. It was therefore proposed that interaction of Endoglin and ALK1 might enhance binding affinity towards BMP9. However, the study of Baeyens and colleagues [19] was performed under serum-deprived conditions and at a short-term time scale for mechanical stimulation. The goal of the present study was to extend on this study and elucidate the attribution of Endoglin to SS-induced SMAD1/5 activation under serum conditions where BMPs and other growth factors are not limited (such as found in human plasma) and on cells that sufficiently adapted their phenotype to the mechanical stimulus for longer period of time. Surprisingly, we found under those conditions that Endoglin limits, rather than enhances FSS-induced SMAD1/5 activation and transcriptional outcomes via BMP9/10. We observed that loss of Endoglin results in exacerbated SMAD1/5 phosphorylation in FSS-adapted cells cultured in a medium supplemented with 20% FCS or human serum. We assume that under limiting ALK1-ligand concentrations, Endoglin with its large extracellular domain [60] supports the sequestration of free circulating BMPs, such as BMP9 and BMP10, to the cell surface receptor ALK1. At saturating serum concentrations of BMP9 (picomolar range, i.e., 0.5–15 ng/mL) [61]; however, the BMP9/10 high-affinity receptor ALK1 (EC_50_: 50 pg/mL) [9, 62] can bind BMP9 independent of Endoglin cooperativity. Crystallization of the extracellular domain of Endoglin in complex with BMP9 highlighted that BMP9 binds to Endoglin at the type II receptor binding interface, thus only allowing for concomitant binding of type I receptor and Endoglin in the absence of type II receptor [11]. Furthermore, SPR experiments showed that Activin type II receptor B (ActRIIB) is not able to bind to a complex of BMP9 and Endoglin extracellular domain (ECD) [63]. In the same study, the authors observed an inhibitory effect of Endoglin ECD on BMP9/10 signaling in a cell-based assay [63]. Therefore, we postulate that the limiting effect of Endoglin on FSS-mediated BMP9 signaling arises from temporary shielding of the ligand for binding the essential signaling type II receptor by ligand-bound Endoglin (Fig. 5). We provide evidence for another mode of Endoglin engagement in FSS-BMP9-ALK1 signaling where Endoglin protects ECs from exacerbated BMP9-induced SMAD1/5 phosphorylation under physiological, i.e., saturated BMP9 conditions, instead of promoting BMP9-ALK1-SMAD1/5 signaling (Fig. 5). Functionally, we provide evidence that loss of Endoglin leads to activation of the endothelium in HSS as we observed increased adhesion of leukocytes to Endoglin KD cells. This is particularly interesting as Endoglin itself is an adhesion molecule and its ECD was shown to enhance leukocyte transmigration in a transwell assay [64]. Adhesion molecule E-Selectin was found to be upregulated by BMP9 stimulation in human pulmonary ECs [65]. Accordingly, we propose that enhanced SMAD1/5 activation upon Endoglin KD in HSS leads to an upregulation of adhesion molecule P-Selectin which allows for increased adhesion of leukocytes.

In our study, we could show for the first time that LSS favors the formation of Endoglin-ALK1 complexes within an endosomal compartment that we identified as EEA1- and Caveolin-1-positive EEs (Fig. 5). These atheroprone SS-induced EEs act as signaling hotspots that increase endothelial SMAD1/5 signaling intensities beyond physiological levels (Fig. 5). Accordingly, these newly identified signaling EEs occur in significantly higher numbers under LSS as compared to HSS conditions. Early studies in shear-exposed ECs showed that long-term exposure (24 h) of ECs to HSS (12 dyn/cm^2^) leads to the formation of submembraneous stress fibers extending in the direction of flow [66]. Additionally, Caveolin-1 redistributes to the upstream edge of ECs subjected to HSS [67]. Thus, HSS might lead to a translocation of Caveolin-1 to areas of high mechanical tension which impedes internalization via CvME [68]. In support of this, it was observed that an increase in membrane tension, induced by mechanical stress, leads to rapid disassembly of caveolae in human cells [69]. Furthermore, it is known that caveola membrane domains switch upon increased shear force from the liquid-ordered state to the liquid-disordered state [70]. By this mechanism, HSS could limit increased pSMAD1/5 activation from EEs because flow would segregate Endoglin from BMPR complexes already at the plasma membrane. By this, EEs serve as an intracellular compartment contributing to FSS-induced BMP-SMAD1/5 signaling. For repulsive guidance molecules (RGMs), a different set of BMP co-receptors, it was shown that their interaction with BMP ligands is pH dependent [71]. It was proposed that the acidic environment in EEs leads to dissociation of RGMs from BMPs, subsequently allowing for type I receptors to bind the respective epitope on the ligand [72]. Although such pH dependency has not yet been described for Endoglin and BMP9, we propose that the mechanism could be similar with the difference that Endoglin’s dissociation from the ligand would allow for the type II receptor to bind, thereby inducing transphosphorylation and activation of type I by type II receptor (Fig. 5).

Interestingly, similar to what we observed in LSS-exposed HUVECs, static experiments in HUVECs showed a decrease of BMP9-induced SMAD1/5 phosphorylation upon Caveolin-1 knock-down [57]. Moreover, several studies show that a depletion of Caveolin-1 markedly reduces atherogenesis in LDLR^−/−^ and ApoE^−/−^ mice [73–75]. Using a triple knock-out model of LDLR^−/−^, eNOS^−/−^, and Cav-1^−/−^ in mice, Ramirez and colleagues could show that this reduction in atherogenesis was independent of altered nitric oxide production but instead relies on the reduction of low-density lipoprotein transcytosis, fibronectin deposition, and generally inflammation [75]. Interestingly, both fibronectin expression and inflammation are regulated by BMP signaling [7, 76, 77]. Moreover, overexpression of BMP antagonist Matrix Gla protein or addition of BMP inhibitor LDN193189 markedly reduced vascular calcification and inflammation in ApoE^−/−^ and LDLR^−/−^ mice, respectively [14, 18]. We therefore think that the atheroprotective effects of Caveolin-1 knock-out are in part attributed to reduced phospho-SMAD1/5 levels and subsequent transcription of atheroprone or inflammatory genes.

We showed that LSS-induced SMAD1/5 phosphorylation and expression of atheroprone and inflammatory genes are dependent on BMP9/10 but are also necessary for HSS-induced expression of EC markers (Fig. 5). There is contradicting data on BMP9-mediated inflammation. In a mouse model of *bronchopulmonary dysplasia*, which was further confirmed in static EC cell culture, BMP9 was found to protect against inflammation [78]. Additionally, a recent study found that inhibition of BMP9/10 signaling via administration of ALK1-Fc enhances systemic inflammation in a vein graft model in mice [79]. On the other hand, BMP9 induced expression of *SELE* and *VCAM-1* and neutrophil recruitment in lipopolysaccharide (LPS) or TNF-α pre-treated ECs in an ALK1-dependent manner [12, 80]. It seems that BMP9 has two modes of action. First, in healthy, unprimed endothelium, BMP9 acts as a quiescence factor [10] that suppresses inflammation [78, 79]. If primed by inflammatory agents like LPS or TNF-α [12, 80] or atheroprone SS, BMP9 acts as an accelerator of these processes attributing to the progression of vascular diseases. The mechanisms underlying this bimodal action of BMP9/10 could be diverse. It is tempting to speculate that depending on the compartment of SMAD activation and complex formation, i.e., plasma membrane versus EEs, SMADs might form complexes with different co-factors. It was shown that signaling endosomes serve as crosstalk hubs for several transcription factors, including BMP and TGFβ SMADs [81]. Therefore, LSS might induce a set of SMAD-co-factor complexes different than in HSS. Interestingly, a very recent study showed that SMAD proteins interact with membrane lipids via their MH2 domain [82]. The authors showed that SMAD2 preferentially binds to phosphatidylinositol-4,5-bisphosphate (PI(4,5)P_2_) which accumulates at the plasma membrane (PM) rather than at EEs. Depending on the affinity of other SMADs to lipids of the PM versus lipids of EEs, different SMAD complexes could arise in HSS and LSS, regulating a different set of target genes. Moreover, the activation of LSS-dependent transcription factors by the preceding stimulus (e.g., atheroprone flow) likely primes chromatin for the binding of SMADs by opening promotor or enhancer regions at putative SMAD target genes that would not be accessible in HSS.

Elucidating the role of BMP signaling in regulating vascular homeostasis and activation is of great importance since severe vascular diseases like PAH and HHT emerge from de-regulation of BMP/TGFβ signaling. Interestingly, for both diseases, PAH and HHT, underlying mutations in ALK1 and Endoglin were described [83, 84]. It was shown additionally that Caveolin-1 plays a crucial role in receptor localization and regulation of downstream SMAD1/5 signaling in PAH [85, 86]. Moreover, the contribution of altered FSS to disease progression of PAH or HHT patient’s blood vessels is discussed [87, 88].

## Conclusions

In summary, we show that Endoglin is necessary to limit levels of BMP9/10-induced SMAD1/5 phosphorylation which protects FSS-exposed ECs against the expression of inflammatory marker *EDN1*. We found that Endoglin localizes to Caveolin-1-positive signaling EEs. This newly identified EE compartment is crucial for LSS-induced increase in atheroprone BMP signaling. Further investigations on BMP signaling crosstalk with FSS mechanobiology are needed, especially considering the role of aberrant BMP signaling in vascular diseases like HHT and PAH. Since depletion of Caveolin-1 leads to a reduction of SMAD1/5, Caveolin-1, rather than ALK1 or BMP9, could be an interesting target for the treatment of vascular diseases, especially in early atherosclerosis [75]. However, since Caveolin also acts as a sensor of altered FSS [56], EC response to flow would need to be characterized carefully in this scenario. Additionally, there is a strong need for further investigating BMP/TGFβ signaling in long-term flow-adapted ECs. Our data here reveal that long-term atheroprone fluid shear stress regulates ALK1-Endoglin-SMAD signaling from the EE.

## Methods

### Cell culture

HUVECs were isolated as described before [89, 90]. Briefly, umbilical cords were placed under sterile conditions in Petri dishes. To remove the remaining blood, umbilical veins were rinsed twice with Hank’s buffered salt solution (HBSS). After removing HBSS, one end of the cords was sealed and the umbilical veins were filled with 10 mL of collagenase type II (1 U/mL, Biochrom KG, Berlin, Germany). The digestion was carried out at 37 °C for 15 min in the incubator. Detached cells were released by flushing the veins twice with HBSS. The mixture was centrifuged at 1200 rpm for 5 min and the cell pellets were resuspended in medium M199. HUVECs were subcultured on gelatin-coated tissue culture ware (Greiner Bio-One) in M199 medium (Sigma-Aldrich; with Earle’s salts and NaHCO_3_) supplemented with 20% fetal calf serum (FCS) (Biochrom) or 20% human serum (biosera), 50 μg/mL endothelial cell growth supplement (Corning), 25 μg/mL heparin, 2 mM l-glutamine, 100 U/mL penicillin, and 0.1 mg/mL streptomycin at 37°C and 5% CO_2_ (full medium). HUVECs were used between passages two to five from isolation. For experiments with serum limitation, HUVECs were placed in an M199 medium containing 25 μg/mL heparin, 2 mM l-glutamine, 100 U/mL penicillin, and 0.1 mg/mL streptomycin at 37°C and 5% CO_2_ (starvation medium). Cells were transferred to a starvation medium 4 h prior to the onset of the experiment. HAoECs (Cell Applications) were cultured in endothelial cell growth medium 2 (Promocell) supplemented with 2% FCS and used at passage 4. All cells were tested negative for mycoplasma contamination.

### siRNA transfections

siRNAs were purchased from Dharmacon (ON-TARGET plus Non-targeting siRNA #1, ON-TARGET plus Human ENG siRNA-SMARTpool, ON-TARGET plus Human CAV1 siRNA-SMARTpool). HUVECs were transfected using Lipofectamine2000 (Thermo Fisher Scientific) with 100 nM siRNA in 6-well plates according to the manufacturer’s instructions. Cells were transferred to μ-Slides (ibidi GmbH) 24 h after transfection and used for experiments 48 h after transfection.

### Application of fluid shear stress

Cells were seeded on gelatin-coated μ-Slide I 0.4 Luer (ibidi GmbH) at 2.5 × 10^6^ cells/mL and cultured for 48 h to reach confluency in the full medium before the experiment with a daily medium exchange. Shear stress was applied using the ibidi pump system (ibidi GmbH) with the associated software (V. 1.5.4). Two pump systems were used to allow simultaneous application of LSS (1 dyn/cm^2^) and HSS (30 dyn/cm^2^). For long-term experiments, cells were allowed to adapt to increasing levels of shear stress in an adaption phase (2.5 h: 2.5 dyn/cm^2^ for 60 min, 5 dyn/cm^2^ for 30 min, 10 dyn/cm^2^ for 30 min, 20 dyn/cm^2^ for 30 min, 30 dyn/cm^2^ for 24 h). Static controls were seeded in silicone forms with the same area as μ-Slides and cultured in 10-cm dishes. For LSS and HSS, we used WHITE and YELLOW-and-GREEN perfusion sets (ibidi GmbH), respectively.

### Inhibitor treatment

For inhibitor experiments, ALK1-Fc (50 ng/mL, R&D Biosystems) was added to a full medium and incubated at 37°C for 30 min prior to application on the cells.

### Leukocyte adhesion assay

Human THP-1 monocyte cells were cultured in RPMI 1640 medium (Gibco; Thermo Fisher Scientific, Inc., MA, USA) containing 10% fetal bovine serum, 2 mM l-glutamine, 100 U/mL penicillin, and 100 μg/mL streptomycin (Gibco; Thermo Fisher Scientific, MA, USA).

For monocyte adhesion experiments, siRNA-transfected HUVECs were seeded into 0.4 μ-Slide I Luer flow chambers (ibidi GmbH, Gräfelfing, Germany) at confluence. Cells were exposed to laminar shear stress of 30 dynes/cm^2^ for 24 h prior to monocyte rolling. THP-1 monocytes were labeled with 1,1′-dioctadecyl-3,3,3′,′tetramethylindo-carbocyanine fluorescent dye (DiI; Invitrogen, Carlsbad, USA) for 15 min at 37°C and washed with PBS. Endothelial monolayers were then perfused with 10^6^ labeled THP-1 cells for 30 at 37°C at 30 dynes/cm^2^. Non-adherent cells were gently washed off with PBS and cells were fixed with 4% paraformaldehyde for 15 min at room temperature. The number of adhered THP-1 cells on HUVECs was assessed by a fluorescence phase-contrast microscope (Biorevo; BZ-9000; Keyence, Osaka, Japan) and quantified using ImageJ Software (Image J, NIH, MD, USA).

### Immunofluorescence

For immunofluorescence stainings, cells were fixed using 4% paraformaldehyde for 10 min and were permeabilized in 0.3% Triton X-100 for 10 min. After blocking with 3% bovine serum albumin (BSA, Carl-Roth GmbH, Karlsruhe, Germany) in PBS for 1 h, primary antibodies were added in 3% BSA in PBS overnight at 4°C. Cells were subsequently washed, incubated with secondary antibodies and DAPI and Phalloidin for 1 h, washed again, and mounted with ibidi Mounting Medium (ibidi GmbH). Images were captured on a Leica TCS SP8 confocal microscope equipped with a 405-nm diode laser, 488-nm argon laser, 561-nm diode laser, and 633nm HeNe laser using a 20× (20×/0.75 HC PL APO Imm Corr WD 0.68 mm) or 63× (63×/1.4 HC PL APO CS2 WD 0.14 mm) oil immersion objective or an expert line Abberior STED microscope equipped with 488-, 561-, and 640-nm diode lasers with a 100× (UPlanSApo 100×/1.40 WD 0.17 mm) immersion oil objective. Antibodies and molecules used were VE-Cadherin XP (2500, Cell Signaling Technology, 1:200), Endoglin (AF 1097, R&D systems, 1:200), Caveolin-1 XP (3267, Cell Signaling Technology, 1:200), EEA1 (610456, BD Transduction Laboratories, 1:200), SMAD1 (ab53745, abcam), Phalloidin (SC-363797, Santa Cruz Biotechnology, 1:1000), and DAPI (D9542, Sigma-Aldrich, 1:500). Secondary antibodies were goat α mouse Alexa594 (A11020, Invitrogen, 1:300), goat α mouse Alexa488 (A11001, Invitrogen, 1:300), goat α rabbit Alexa488 (A11034, Invitrogen, 1:300), goat α rabbit Alexa594 (A11012, Invitrogen, 1:300), donkey α goat Alexa488 (A11055, Invitrogen, 1:300), and goat α rabbit Alexa647 (A21244, Invitrogen, 1:300).

### Labeled BMP9

For fluorescent labeling of BMP9, NHS-SiR-d12 (generously provided by Dr. Johannes Broichhagen, Forschungsinsitut für Molekulare Pharmakologie, Berlin-Buch, Germany, method described in [58]) was taken up in DMSO (final concentration of 1 mM). Lyophilized BMP9 (Peprotech, #120-07) was reconstituted in sodium bicarbonate buffer (0.2 M, pH 8.3) (reaction buffer) to obtain the final concentration (2 μM). NHS-SiR-d12 was then added to the reconstituted BMP9 ligands at a 5-fold excess. The mixture was allowed to incubate at room temperature for 4 h. Meanwhile, a Amicon Ultra 10-kDa molecular weight cutoff column (0.5 mL) (Merck Millipore, UFC501024) was calibrated with reaction buffer and centrifuged at 14,000 × g at 4 °C without drying the column. The reaction mixture was then gently applied onto the pre-calibrated column and centrifuged for 4 min, while never allowing the column to dry. Subsequently, the column was gently washed with reaction buffer (500 μL) and centrifuged as described before. This washing procedure was repeated twice. Afterwards, sterile Millipore H_2_O (50 μL) was added onto the column, before the column was inverted and put in a fresh elution tube and centrifuged as described above. The column was rinsed with sterile Millipore H_2_O (30 μL) and eluted as in the previous step. Protein concentration was determined with a Nano Drop 2000 spectrophotometer (ThermoFischer).

For experiments, HUVECs were pre-flowed for 24 h before flow slides were detached from the pump system and 600 nM BMP9 SiR-d12 was added on the cells in an M199 medium supplemented with 25 μg/mL heparin, 2 mM l-glutamine, 100 U/mL penicillin, and 0.1 mg/mL streptomycin. Cells were allowed to take up BMP9 SiR-d12 for 10 min at 37 °C prior to fixation. Images were acquired using 640-nm laser excitation at the expert line Abberior STED microscope (see the “Immunofluorescence” section).

### Image analysis

Alignment of actin stress fibers was performed with FiberFit software [91]. Counting of Caveolin vesicles and Endoglin-positive Caveolin vesicles was performed in ImageJ using a self-written script (https://github.com/Habacef/Atheroprone-FSS-regulated-ALK1-Endoglin-SMAD-signaling-originates-from-early-endosomes [92]). In short, EEA1 or Caveolin-rich vesicles were identified via AnalyzeParticles function. Caveolin vesicles were translated into a mask and Endoglin intensity in that mask was measured. In R, Endoglin intensity in Caveolin vesicles was compared to the mean Endoglin intensity of the image and Caveolin vesicles were marked as Endoglin positive if it was at least 15% higher than mean Endoglin intensity. For the exact parameters used, refer to the script. Intensity profiles were measured in ImageJ. For EEA1 and SMAD1 enrichment, first, background subtraction and thresholding was performed in ImageJ and subsequently Cav-1- and Endoglin-positive vesicles were screened for EEA1/SMAD1 signal in R.

### Western blot

Cells were lysed in RIPA buffer (50 mM Tris, 150 mM NaCl, 1% Triton X-100 (v/v), 1% IGEPAL (v/v), 0.1% SDS), and protein concentration was measured with BCA test (Thermo Fisher Scientific). Subsequently, lysates were supplemented with Laemmli buffer [93], boiled at 95 °C for 10 min prior to separation on 10% SDS–PAGE gels. Proteins were transferred onto nitrocellulose membranes (neoLab Migge Laborbedarf-Vertriebs GmbH, Heidelberg, Germany). Membranes were blocked in 5% w/v BSA (Sigma-Aldrich) in TBST and then incubated with primary antibodies overnight at 4 °C. The next day, membranes were incubated with goat-α-rabbit-HRP, goat-α-mouse-HRP (1:10,000, Dianova, Hamburg, Germany), or mouse-α-goat (1:10,000, Santa Cruz Biotechnology), prior to detection with WesternBright Quantum ECL HRP substrate (advansta, Menlo Park, USA) using a Fusion-FX7 (Vilber Lourmat, Eberhardzell, Germany). Primary antibodies used were Endoglin (AF 1097, R&D systems), Caveolin-1 XP (3267, Cell Signaling Technology), EEA1 (610456, BD Transduction Laboratories), pSMAD1/5 (9516, Cell Signaling Technology), and GAPDH (2118, Cell Signaling Technology). The concentration of primary antibodies was 1:1000 in 3% BSA.

### Quantitative real-time PCR

Cellular RNA was isolated using the NucleoSpin RNA XS isolation kit (Macherey-Nagel, Düren, Germany) according to the manufacturer’s instructions. 0.5 to 1 μg total RNA was reversely transcribed by incubating it with random primers (100 pmol μL^–1^, Invitrogen, Carlsbad, USA) and M-MuLV reverse transcriptase enzyme (200,000 U mL^–1^, New England Biolabs, Ispwich, USA) was added per sample. RT-PCR was performed using a StepOnePlus Real-Time PCR System (Thermo Fisher Scientific) with specific primers for the genes listed in Additional file 1: Table S1. Reactions were performed in triplicates in MicroAmp Optical 96-well reaction plates (Thermo Fisher Scientific) using SYBR Green PCR Master Mix (Invitrogen) or Luna PCR Master Mix (New England Biolabs). Fold induction was calculated by comparing relative gene expression to the housekeeping gene RSP9 using the ∆∆CT method.

### RNAseq experiment

Shear stress was applied for 24 h in full medium. For each experimental condition (static, HSS, LSS), three biological replicates were analyzed. After initial quality control using Agilent’s Bioanalyzer, sequencing libraries were prepared from 500 ng of total RNA per sample following Roche’s stranded “KAPA RNA HyperPrep” library preparation protocol for single indexed Illumina libraries: First, the polyA-RNA fraction was enriched using oligo-dT-probed paramagnetic beads. Enriched RNA was heat-fragmented and subjected to first-strand synthesis using random priming. The second strand was synthesized incorporating dUTP instead of dTTP to preserve strand information. Afterwards, A-tailing Illumina sequencing-compatible adapters were ligated. Following bead-based clean-up steps, the libraries were amplified using 10 cycles of PCR. Library quality and size were checked with qBit, Agilent Bioanalyzer, and qPCR. Sequencing was carried out in biological triplicates on an Illumina HiSeq 4000 system in SR75bp mode (single read, 75 bp read length) yielding between 46 and 69 million fragments per sample.

### RNAseq data analysis

Single-end, 75-bp reads from Illumina sequencing were mapped to the reference genome (hg19) using the STAR mapper [94] (splice junctions based on RefSeq; options: --alignIntronMin20 --alignIntronMax500000 --outFilterMismatchNmax 10). Differential gene expression was ascertained using the DESeq2 package [95]. The cutoff for significantly altered gene expression was a fold change of < 0.66 or > 1.5 with an adjusted *p*-value < 0.05. Data visualization was performed with RStudio [96] environment for R [97] using the following packages: ggplot2 [98], dplyr [99], ggrepel [100], and pheatmap [101].

For analysis of public data from [41] (GSE103672 [43]), raw FASTQ files were uploaded to the Galaxy platform [102] and processed using STAR [94] (reference genome hg38), StringTie [103], and DESeq2 [95].

### Statistical analysis

For statistical analysis, we used GraphPad Prism version 9.3.0 for Windows (GraphPad Software, San Diego, CA, USA).

### Graphical schemes and figures

Figures were created with BioRender.com.

## Supplementary Information

**Additional file 1: Supplementary Figs. S1-S5.** and primer sequences used in qPCR experiments. **Figure S1.** RNAseq analysis and HAoEC flow marker validation. **Figure S2.** Validation of used flow set up by comparison with existing data. **Fig. S3.** Human Serum experiment and additional flow regulated genes. **Figure S4.** Expression data of selected genes in Endoglin knock-down. **Figure S5.** Caveolin-1 positive early endosomes are signaling hotspots for FSS induced BMP signaling in HAoECs. **Table S1.** Primers used for quantitative PCR analysis.

**Additional file 2.** RNAseq csv file: RPKM values.

**Additional file 3.** RNAseq csv file: Differential expression values of LSS vs. HSS.

**Additional file 4. **Uncropped Western Blot images.

## Data Availability

RNA sequencing raw data produced for this study are publicly available under the accession number GSE135312 [34] at the National Center for Biotechnology Information advances science and health; Gene Expression Omnibus platform under the following link: https://www.ncbi.nlm.nih.gov/geo/query/acc.cgi?acc=GSE211662 [104]. We provide csv files with RNAseq RPKM values and differential gene expression (from DeSeq2) as additional files along with the manuscript. Code for image analysis is available from GitHub: https://github.com/Habacef/Atheroprone-FSS-regulated-ALK1-Endoglin-SMAD-signaling-originates-from-early-endosomes [92]. Public data analyzed are available from Gene Expression Omnibus platform with accession number GSE103672 [43].
