# Supplementary material for: Atheroprone fluid shear stress-regulated ALK1-Endoglin-SMAD signaling originates from early endosomes
Source: BMC Biol. 2022 Sep 28;20:210. doi: 10.1186/s12915-022-01396-y (PMC9520843; doi:10.1186/s12915-022-01396-y)
Supplement: Supplementary file 1 — Additional file 1: Supplementary Figs. S1-S5. and primer sequences used in qPCR experiments. Figure S1. RNAseq analysis and HAoEC flow marker validation. Figure S2. Validation of used flow set up by comparison with existing data. Fig. S3. Human Serum experiment and additional flow regulated genes. Figure S4. Expression data of selected genes in Endoglin knock-down. Figure S5. Caveolin-1 positive early endosomes are signaling hotspots for FSS induced BMP signaling in HAoECs. Table S1. Primers used for quantitative PCR analysis. [file 12915_2022_1396_MOESM1_ESM.pdf]

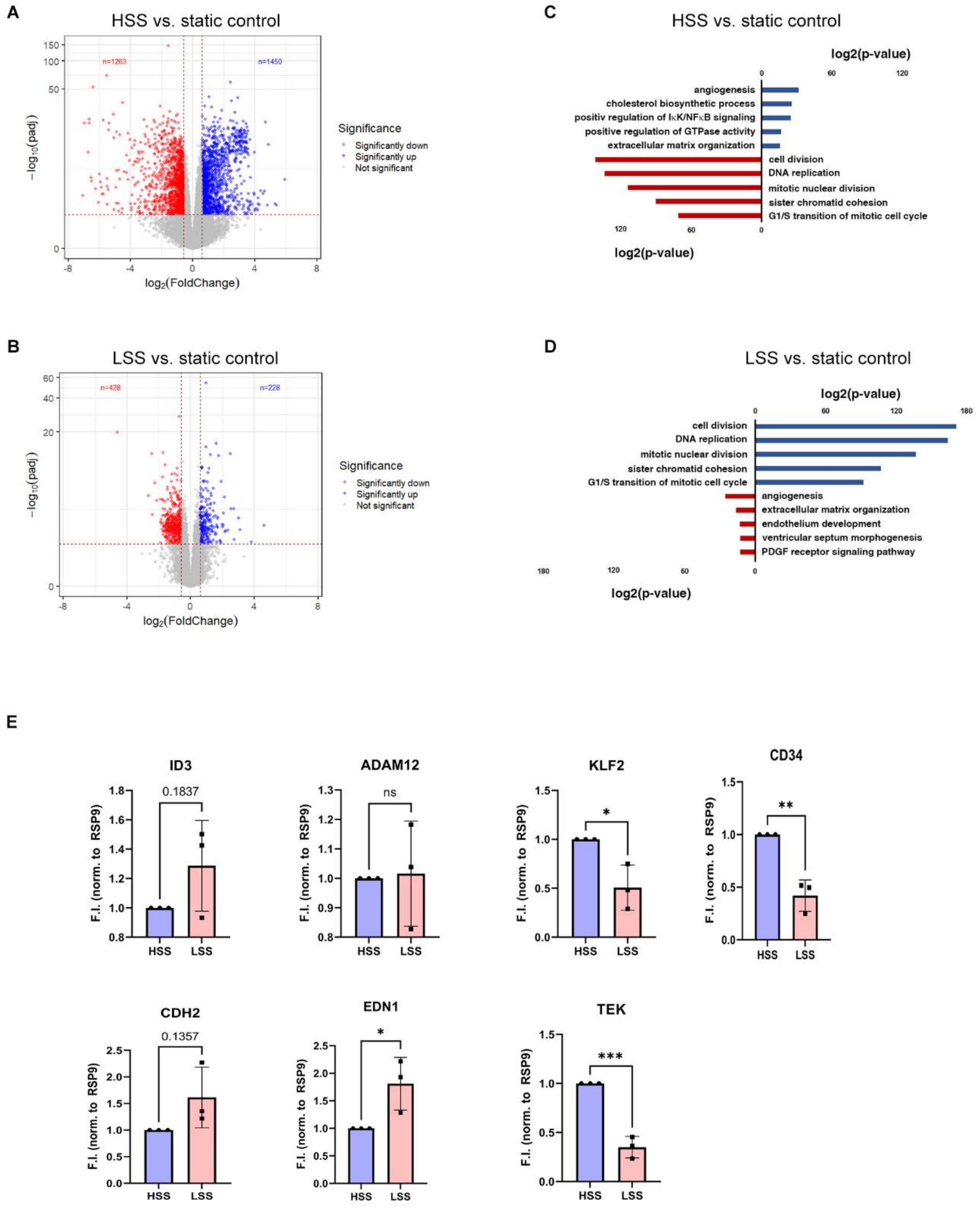

Figure S1: RNAseq analysis and HAoEC flow marker validation. **A/B** Volcanoplot of all DEGs (adjusted p-value < 0.05, log2FC > |0.585|, dotted lines) in HSS/ LSS versus static. **C/D** Gene Ontology (Biological Process) analysis of DEGs from HSS/ LSS versus static. **E** qPCR expression data from HAoECs (n=3 biological replicates) upon exposure to HSS/ LSS. Statistical significance against HSS control was calculated using 2-way ANOVA and Šídák's post-hoc test

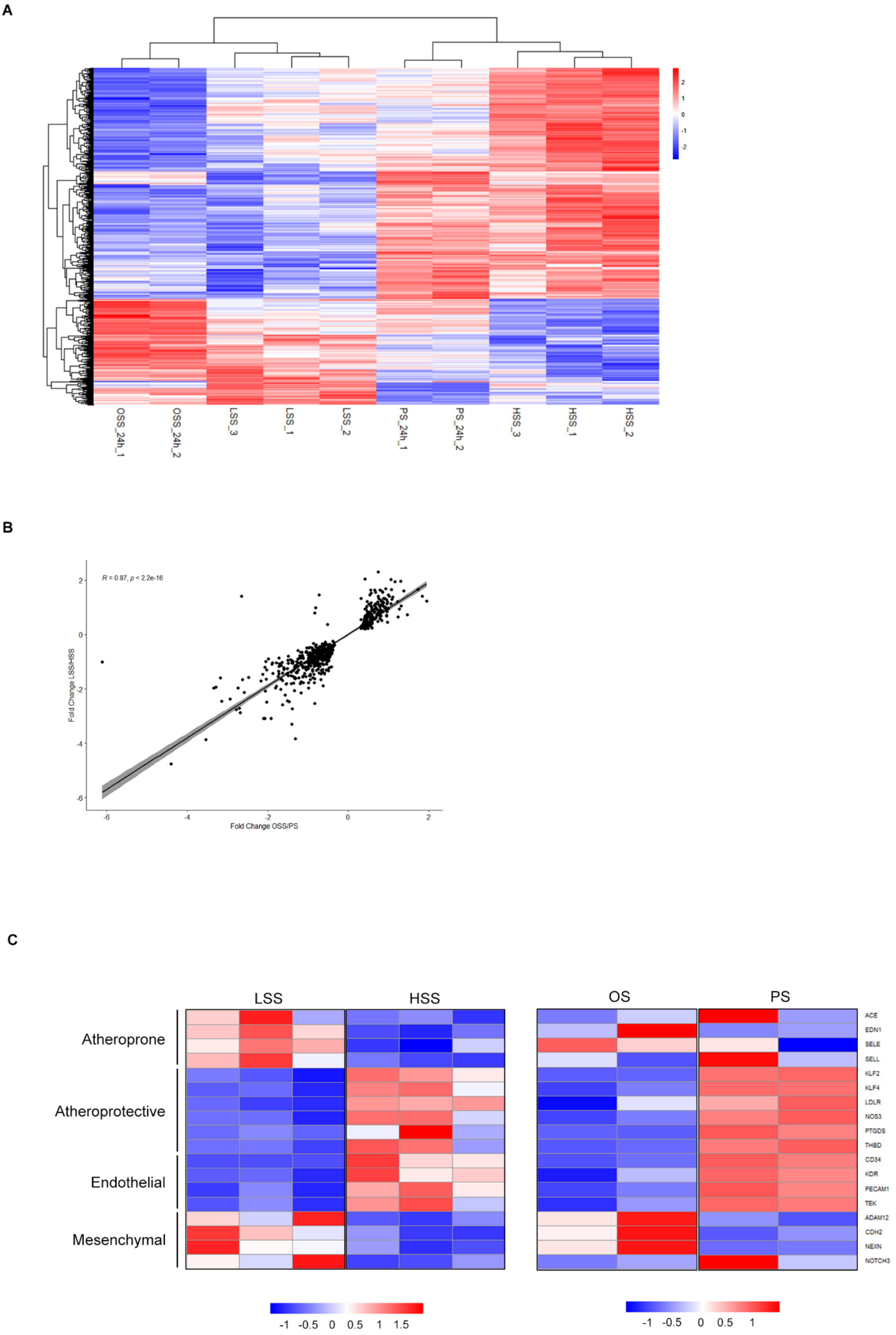

Figure S2: Validation of used flow set up by comparison with existing data. A Heatmap **c**omparing RNA seq data (z-scores) from this publication with data available from GSE103672. All genes are differentially expressed in both datasets (adjusted p-value < 0.05, log2FC > |0.585|). Rows (genes) and columns (samples) are hierarchically clustered. **B** Correlation plot comparing expression of DEGs from RNAseq data of this publication with data available from GSE103672. **C** Heatmaps of selected marker genes. Comparison of RNA seq data from this publication with data available from GSE103672. Genes are DEGs for the left panel. Genes from the right panel (GSE103672) were extracted independent of p-value or fold change. Color-coding shows Z-scores. Z-scores were calculated separately foreach of the two RNAseq experiments.

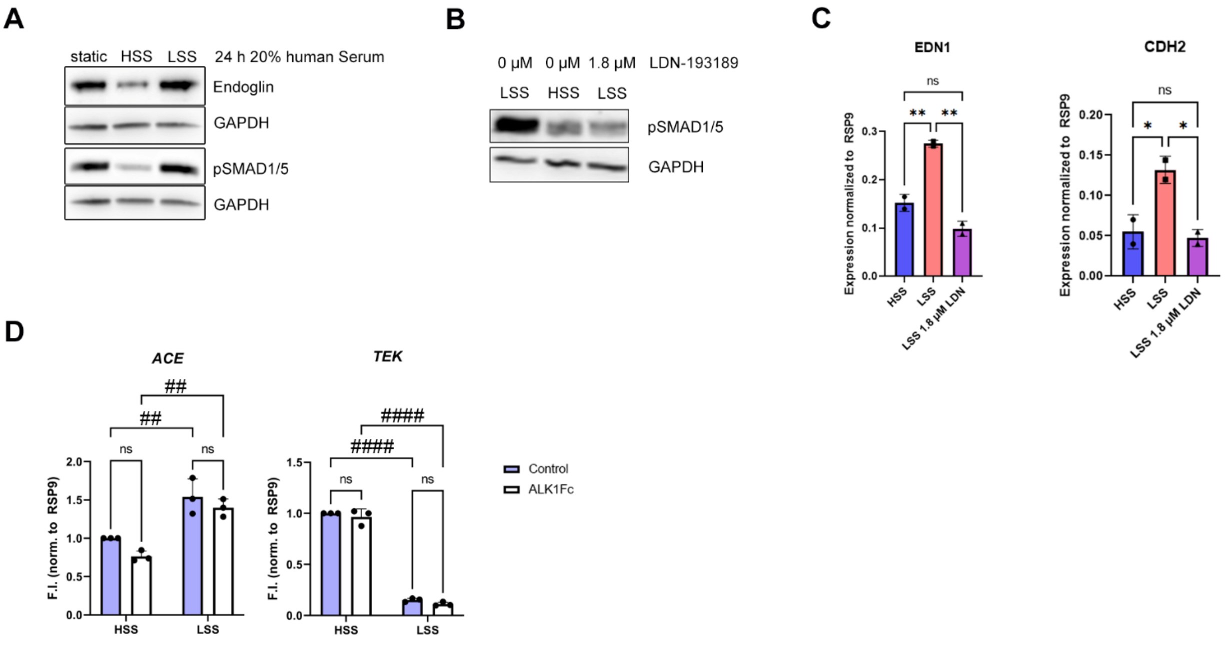

Figure S3: Human Serum experiment and additional flow regulated genes. A Immunoblot using antibodies specific against pSMAD1/5 and Endoglin of HUVECs exposed to HSS and LSS in the presence of human serum instead of FCS. pSMAD1/5 and Endoglin levels are similar to FCS experiments. **B** Immunoblot using antibodies specific against pSMAD1/5 and GAPDH of HUVECs exposed to HSS and LSS in the presence of BMPRI inhibitor LDN-193189. **C** qPCR expression data for selected genes in HUVECs exposed to HSS and LSS in the presence of BMPRI inhibitor LDN-193189. **D** Quantitative PCR showing gene expression of selected markers after 24 h FSS application in absence/ presence of 50 ng/ mL ALK1-Fc. Presented genes are dependent on FSS but not BMP9. Data is presented as mean ± SD from 3 independent experiments. Statistical significance against HSS control was calculated using 2-way ANOVA and Šídák's post-hoc test, $^{*}p \leq 0.05$, $^{**}p \leq 0.01$, $^{***}p \leq 0.001$, $^{****}p \leq 0.0001$, ns means not significant ($p > 0.05$). Statistical significance in between groups (HSS vs. LSS) was calculated using 2-way ANOVA and Šídák's post-hoc test, $^{\#\#\#\#}p < 0.0001$, $^{\#\#}p < 0.01$.

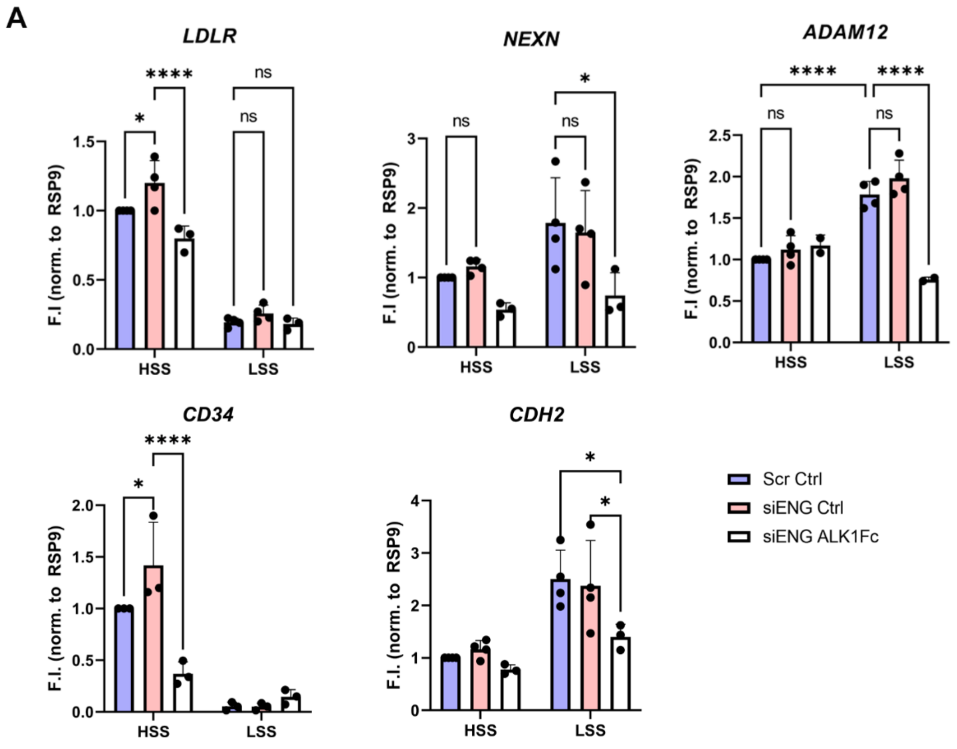

Figure S4: Expression data of selected genes in Endoglin knock-down. A qPCR expression data of selected genes from HUVECs exposed to HSS and LSS upon Endoglin KD and simultaneous addition of 50 ng/ml ALK1Fc. Statistical significance against HSS control was calculated using 2-way ANOVA and Šídák's post-hoc test. *p ≤ 0.05, **p ≤ 0.01, ***p ≤ 0.001, ****p ≤ 0.0001, ns means not significant (p >0.05).

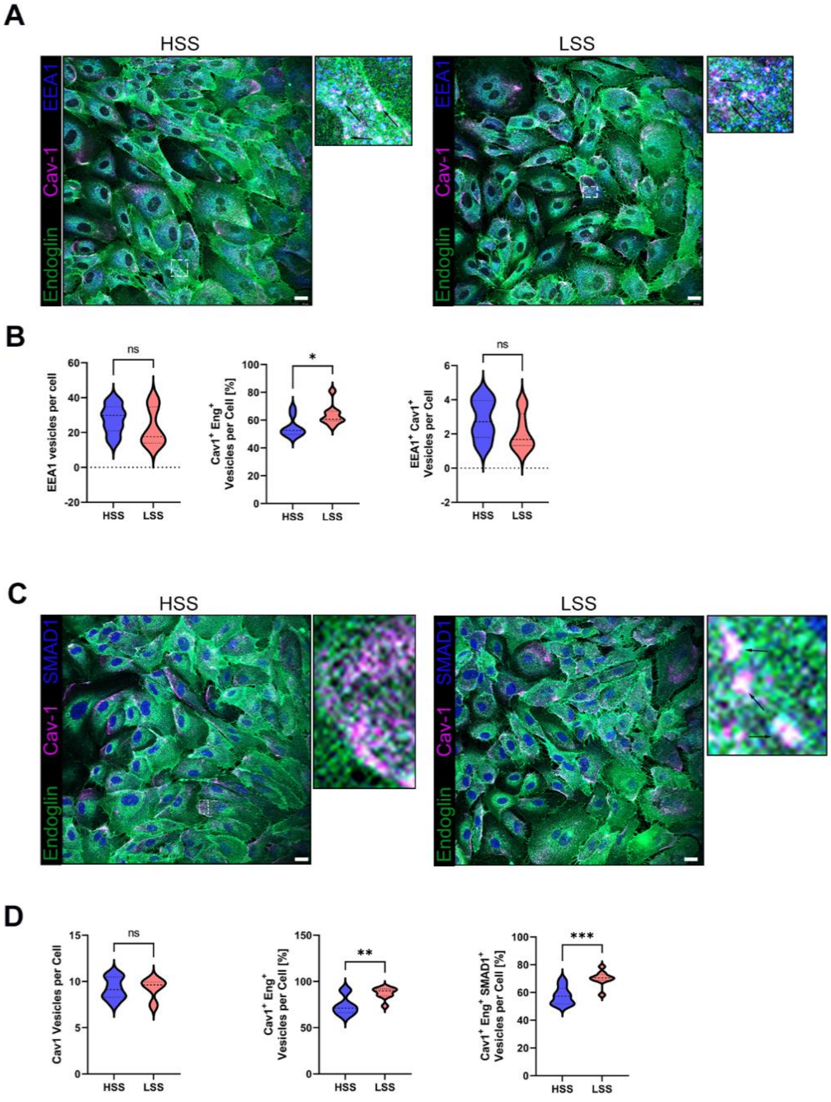

Figure S5: Caveolin-1 positive early endosomes are signaling hotspots for FSS induced BMP signaling.

A Confocal images of Endoglin, Caveolin-1 and EEA1 in aortic endothelial cells exposed to HSS or LSS. Insets show magnified regions. Arrows indicate Endoglin and EEA1 positive Caveolin vesicles. Scale bars 20 µM. **B** Quantification of number of EEA1 vesicles (left), Endoglin positive Caveolin-1 vesicles (middle) and Endoglin and EEA1 double positive Caveolin-1 vesicles (right). Counting was performed using a self-written ImageJ script (see materials and methods for details). **C** Confocal images of Endoglin, Caveolin-1 and SMAD1 in aortic endothelial cells exposed to HSS or LSS. Insets show magnified regions. Arrows indicate Endoglin and SMAD1 positive Caveolin vesicles. Scale bars 20 µM. **D** Quantification of number of EEA1 vesicles (left), Endoglin positive Caveolin-1 vesicles (middle) and Endoglin and SMAD1 double positive Caveolin-1 vesicles (right). Counting was performed using a self-written ImageJ script (see materials and methods for details). Significances were calculated using unpaired Student's t-test. *$p \leq 0.05$, **$p \leq 0.01$, ***$p \leq 0.001$, ****$p \leq 0.0001$, ns means not significant ($p > 0.05$).

Table S1: Primer sequences

Primer name	orientation	Sequence
TEK	fw	GCCTTCCAAAACGTGAGGG
TEK	rev	TGATGCGCGCCTTAAGAACT
EDN1	fw	GGGATCAGAGCAGGAGCATC
EDN1	rev	CTCCACCCCTGTGTGGAATC
CD34	fw	CACCCTGTGTCTCAACATGG
CD34	rev	GGCTTCAAGGTTGTCTCTGG
CDH2	fw	AGGCTTCTGGTGAAATCGCA
CDH2	rev	TGCAGTTGCTAAACTTCACATTG
ACE	fw	TCTGGCAGAACTTCACGGAC
ACE	rev	TTAGCAGGGCGTTGTACTGC
ID3	fw	CTTCCGGCAGGAGAGGTT
ID3	rev	AAAGGAGCTTTTGCCACTGA
SLUG	fw	CACACGGGGGAGAAGCCTTT
SLUG	rev	ATTGCGTCACTCAGTGTGCT
NEXN	fw	TCCACGCGGAAAGAAGTACC
NEXN	rev	TGAAGAAAGCAGAATCTCAGCC
ADAM12	fw	CGCTCGAAATTACACGGGTC
ADAM12	rev	ACGCTTTTCAGCTTCTTCGC
LDLR	fw	GAGGGCTCTGTCCATTGTCC
LDLR	rev	GACCATCTGTCTCGAGGGGT
SELP	fw	GCGGTGGCTTCTACGATAGG
SELP	rev	TTCATGGGTGTTTATGGAAACCTTA

fw - forward; rev - reverse

Table S1: Primers used for quantitative PCR analysis.
