# Supplementary material for: Atheroprone fluid shear stress-regulated ALK1-Endoglin-SMAD signaling originates from early endosomes
Source: BMC Biol. 2022 Sep 28;20:210. doi: 10.1186/s12915-022-01396-y (PMC9520843; doi:10.1186/s12915-022-01396-y)

Figure 1:

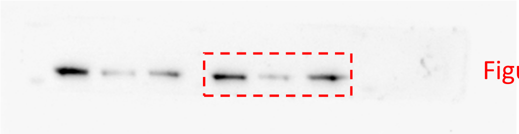

Figure 1E: Endoglin

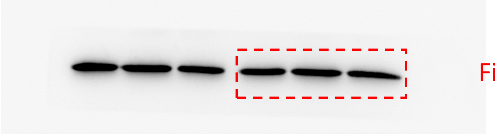

Figure 1E: GAPDH

Figure 2:

Figure 2 A: pSMAD1/5

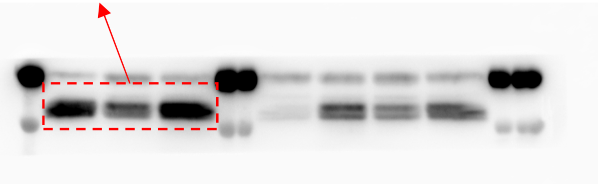

Figure 2 A: GAPDH

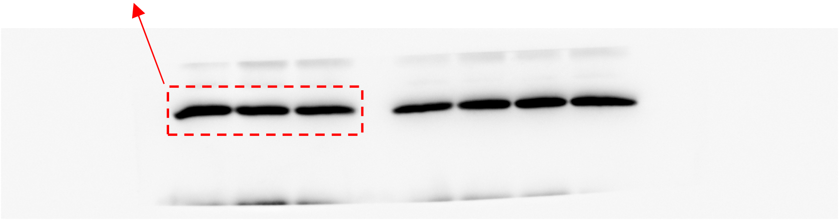

Figure 3A/B:

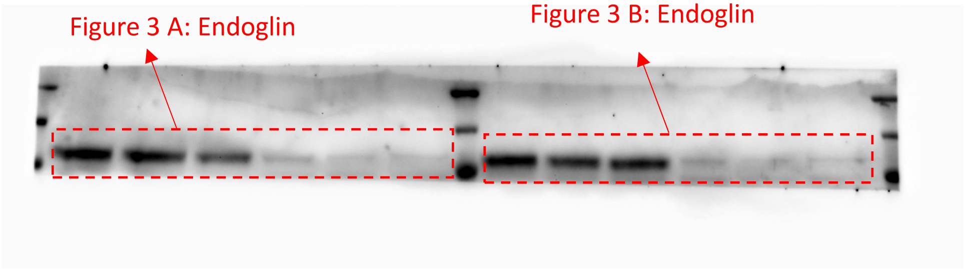
Figure 3 A: Endoglin
Figure 3 B: Endoglin

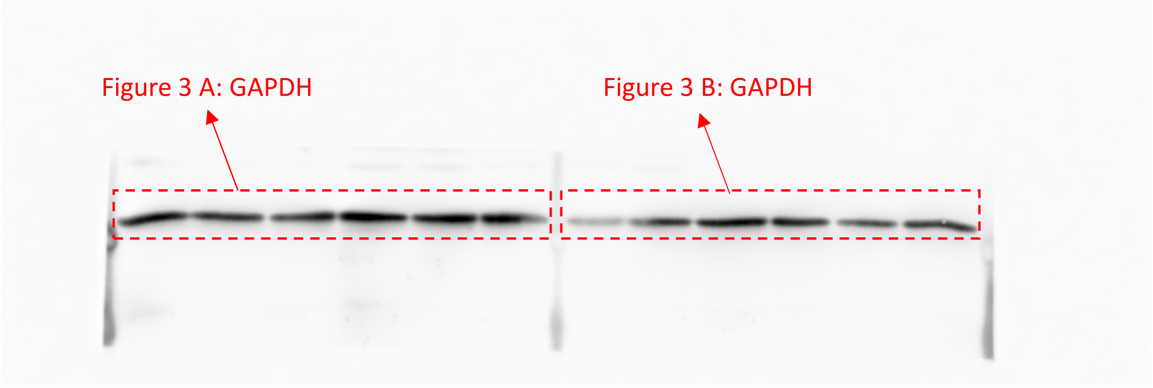
Figure 3 A: GAPDH
Figure 3 B: GAPDH

Figure 3C:

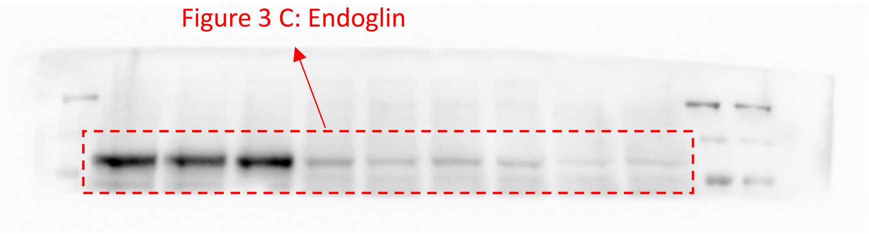

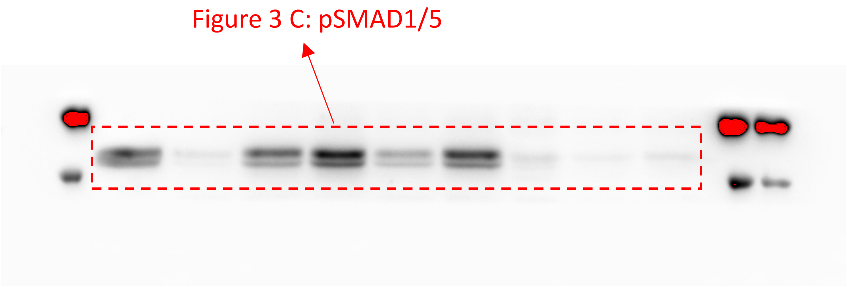

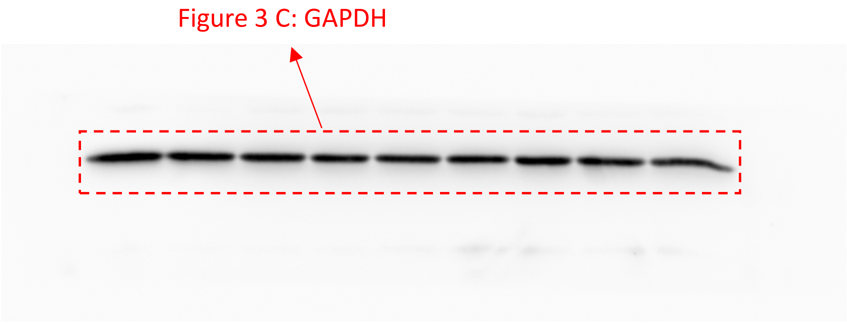

Figure 4:

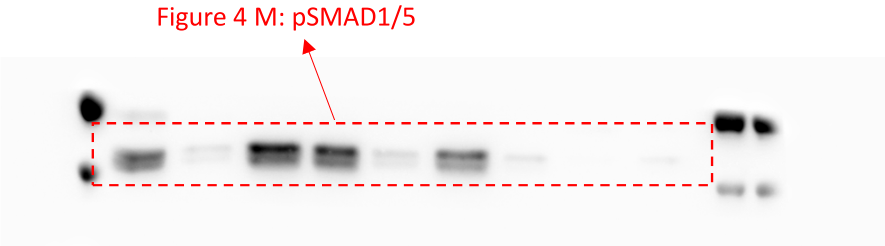

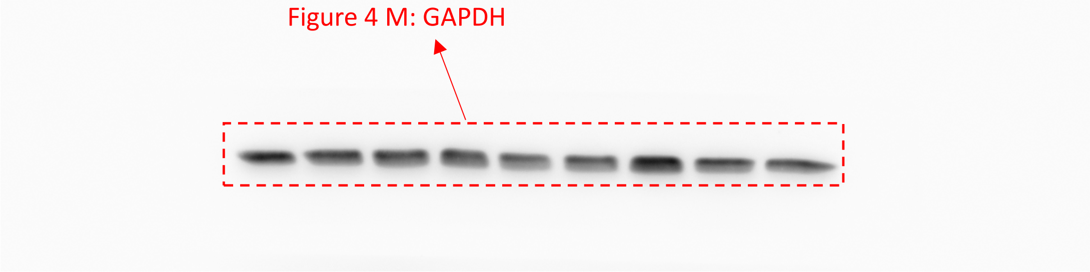

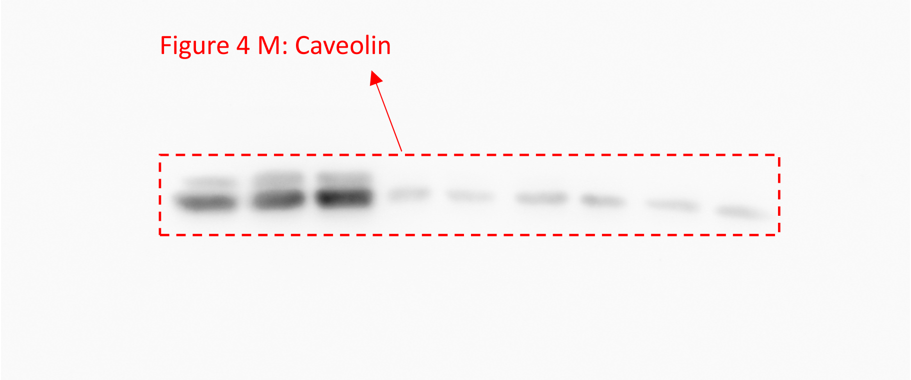

Figure S3A:

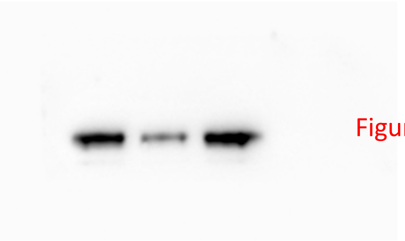

Figure S3 A: Endoglin

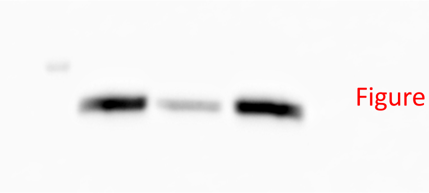

Figure S3 A: pSMAD1/5

Figure S3 A: GAPDH (for pSMAD1/5)

Figure S3 A: GAPDH (for Endoglin)

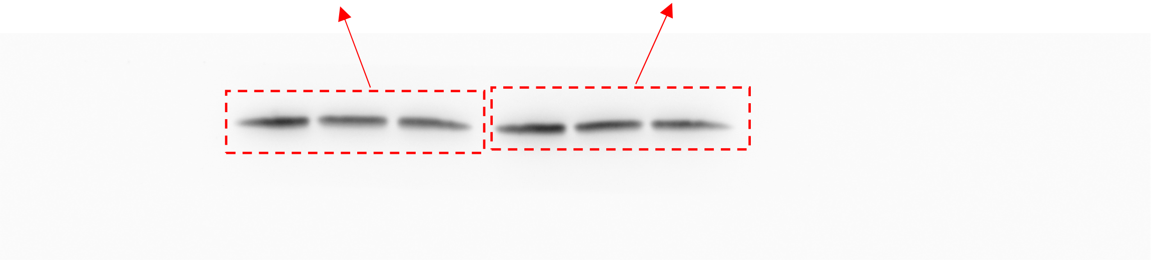

Figure S3B:

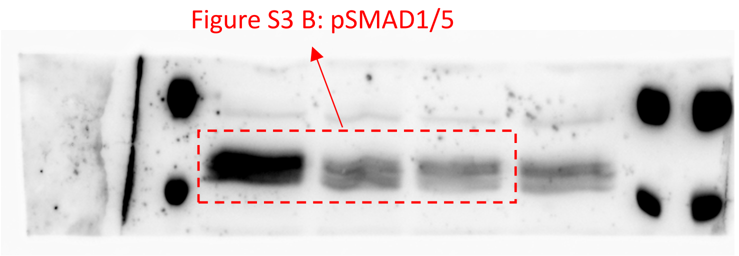

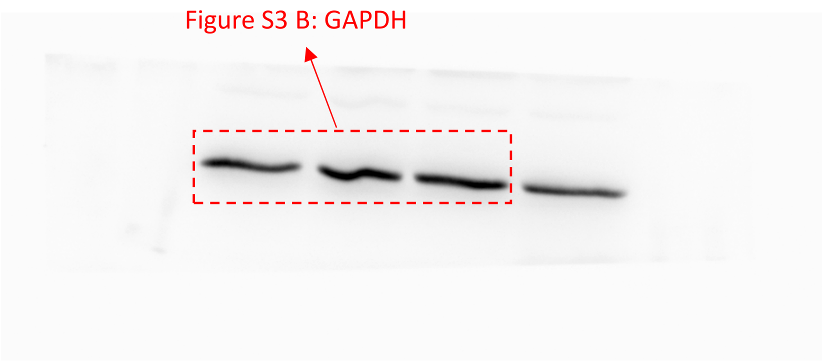

Supplement: Supplementary file 4 — Additional file 4. Uncropped Western Blot images. [file 12915_2022_1396_MOESM4_ESM.pdf]
